# Modelling kidney disease with CRISPR-mutant kidney organoids derived from human pluripotent epiblast spheroids

**DOI:** 10.1038/ncomms9715

**Published:** 2015-10-23

**Authors:** Benjamin S. Freedman, Craig R. Brooks, Albert Q. Lam, Hongxia Fu, Ryuji Morizane, Vishesh Agrawal, Abdelaziz F. Saad, Michelle K. Li, Michael R. Hughes, Ryan Vander Werff, Derek T. Peters, Junjie Lu, Anna Baccei, Andrew M. Siedlecki, M. Todd Valerius, Kiran Musunuru, Kelly M. McNagny, Theodore I. Steinman, Jing Zhou, Paul H. Lerou, Joseph V. Bonventre

**Affiliations:** 1 Division of Renal Medicine, Department of Medicine, Brigham and Women’s Hospital, Harvard Medical School, Harvard Institutes of Medicine Suite 550, 4 Blackfan Circle, Boston, Massachusetts 02115, USA; 2 Division of Nephrology, Department of Medicine, University of Washington School of Medicine, 850 Republican Street, Room S565, Seattle, Washington 98109, USA; 3 Kidney Research Institute, Department of Medicine, University of Washington, 325 Ninth Avenue, Box 359606, Seattle, Washington 98104, USA; 4 Institute for Stem Cell and Regenerative Medicine, University of Washington, Seattle, Washington 98109, USA; 5 Harvard Stem Cell Institute, Harvard University, 7 Divinity Avenue, Cambridge, Massachusetts 02138, USA; 6 Department of Biological Chemistry and Pharmacology, Boston Children’s Hospital, Center for Life Sciences, Harvard Medical School, Room 3103, 3 Blackfan Circle, Boston, Massachusetts 02115, USA; 7 Division of Genetics, Department of Medicine, Brigham and Women’s Hospital, Harvard Medical School, Harvard Institutes of Medicine Suite 823, 4 Blackfan Circle, Boston, Massachusetts 02115, USA; 8 Department of Pediatric Newborn Medicine, Brigham and Women’s Hospital, Harvard Medical School, Boston, Massachusetts 02115, USA; 9 Department of Stem Cell and Regenerative Biology, Harvard University, Sherman Fairchild Biochemistry Building 160, 7 Divinity Avenue, Cambridge, Massachusetts 02138, USA; 10 The Biomedical Research Centre, University of British Columbia, 2222 Health Sciences Mall, Vancouver, British Columbia, Canada V6T 1Z3; 11 Division of Cardiovascular Medicine, Department of Medicine, Brigham and Women’s Hospital, Harvard Medical School, Boston, Massachusetts 02115, USA; 12 Division of Nephrology, Department of Medicine, Beth Israel Deaconess Medical Center, Harvard Medical School, 330 Brookline Avenue DA517, Boston, Massachusetts 02115, USA

## Abstract

Human-pluripotent-stem-cell-derived kidney cells (hPSC-KCs) have important potential for disease modelling and regeneration. Whether the hPSC-KCs can reconstitute tissue-specific phenotypes is currently unknown. Here we show that hPSC-KCs self-organize into kidney organoids that functionally recapitulate tissue-specific epithelial physiology, including disease phenotypes after genome editing. In three-dimensional cultures, epiblast-stage hPSCs form spheroids surrounding hollow, amniotic-like cavities. GSK3β inhibition differentiates spheroids into segmented, nephron-like kidney organoids containing cell populations with characteristics of proximal tubules, podocytes and endothelium. Tubules accumulate dextran and methotrexate transport cargoes, and express kidney injury molecule-1 after nephrotoxic chemical injury. CRISPR/Cas9 knockout of podocalyxin causes junctional organization defects in podocyte-like cells. Knockout of the polycystic kidney disease genes *PKD1* or *PKD2* induces cyst formation from kidney tubules. All of these functional phenotypes are distinct from effects in epiblast spheroids, indicating that they are tissue specific. Our findings establish a reproducible, versatile three-dimensional framework for human epithelial disease modelling and regenerative medicine applications.

## Abstract (web summary)

Generating organized kidney tissues from human pluripotent stem cell is a major challenge. Here, Freedman *et al*. describe a differentiation system forming spheroids and tubular structures, characteristic of these kidney structures, and using CRISPR/Cas9, delete PKD1/2, to model polycystic kidney disease.

## Graphical abstract

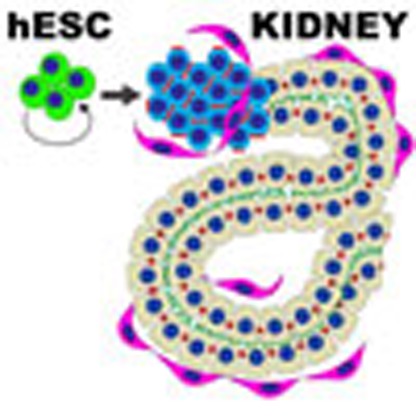

Both undifferentiated stem cells and terminally differentiated somatic cells form epithelia. These can function to establish axes for differentiation in the embryo, or to perform barrier and transport roles in adult organs such as the kidney. Three-dimensional (3D) cell culture *in vitro* is a powerful tool for investigating epithelial morphogenesis, physiology and disease, being readily accessible to microscopic inspection, chemical treatment and experimental manipulation. Studies of epithelial cell lines such as Madin–Darby canine kidney (MDCK) cells have, for instance, revealed polarity and apoptosis pathways contributing mechanistically to lumen formation^1^. Conventional epithelial cell lines, however, are lineage-restricted and lack genetic diversity. As a result, the 3D structures that arise are relatively simple, and it has been challenging to perform controlled comparisons of different epithelia of the same genetic background, or the same epithelia with different genetic backgrounds. Despite these limitations, interest in the cellular microenvironment and 3D culture systems has been increasing steadily, particularly for stem cell applications^2^. There is a significant need for genetically diverse cell culture platforms that accurately reconstitute tissue-specific epithelial function, particularly in humans where species–specific toxicology and disease pathophysiology is of significant biomedical relevance.

Human pluripotent stem cells (hPSCs) are capable of extensive self-renewal and can differentiate into diverse somatic cell types and tissues. hPSCs are also genetically diverse, including thousands of human embryonic stem cell (hESC) and induced pluripotent stem cell (iPSC) lines with patient-specific or gene-targeted mutations^3^,^4^,^5^,^6^. hPSCs have therefore emerged as a powerful and reproducible source of diverse human tissues for disease modelling and regeneration. hPSCs resemble the implantation-stage human epiblast, a tissue that forms the axes for the developing embryo and cannot be studied in living human embryos owing to ethical considerations^2^,^7^,^8^,^9^,^10^. Like the epiblast, hPSCs are epithelial cells, but their polarity, barrier and lumenogenesis characteristics remain very poorly understood. Mouse ESCs (mESCs) were recently shown to form polarized rosettes with small cavities when surrounded by Matrigel extracellular matrix, suggesting the possibility of modelling early amniotic cavity formation in the epiblast^11^. However, because these experiments were performed with mESCs, which more closely resemble the more primitive inner cell mass (ICM) than the epiblast, it remains unclear whether the observed rosettes truly represent epiblast and whether hPSCs could form similar structures^8^,^12^,^13^,^14^,^15^,^16^. Better understanding of human epiblast-stage biology may lead to improvements in the directed differentiation of hPSCs into specific cell types and organoids.

The kidney is an epithelial organ of major interest to the field of regenerative medicine^17^,^18^,^19^,^20^,^21^. Kidney epithelial subsets are highly specialized and their dysfunction can result in a variety of clinical disorders. For instance, polycystic kidney disease (PKD) features cystic expansion of tubular epithelial cells, whereas glomerulopathies involve injury to the podocyte epithelium through which blood is filtered into the tubules^22^,^23^,^24^,^25^,^26^,^27^,^28^. As proof-of-principle for using hPSCs to model kidney disease, we have identified a ciliary phenotype in undifferentiated iPSCs and descendant epithelial cells from PKD patients^17^. Intriguingly, hPSCs have been directed to differentiate *in vitro* into hPSC-derived kidney cells (hPSC-KCs) expressing markers typical of kidney progenitor cells, proximal tubules and podocytes^18^,^19^,^20^,^21^. However, these markers may not be exclusive to the kidney, and no study to date has demonstrated an ability to form renal-like structures *in vitro* and recapitulate a disease-relevant phenotype in hPSC-KCs. Ideally, such phenotypes would be expected to be present in kidney tissues but be absent in other epithelial cells not similarly affected by the disease. Reconstitution of kidney-specific microphysiology and injury/disease states in hPSC-KCs is therefore important to more conclusively identify these epithelia and to advance their translational application.

Here we establish adherent, 3D growth conditions for reconstitution of two distinct epithelial structures, epiblast spheroids and kidney organoids, which arise sequentially in a single continuous culture of hPSCs. Using small molecule treatments and genome-edited hPSCs, we demonstrate that these structures are capable of reconstituting tissue-specific epithelial transport, toxicity responses and disease phenotypes. Our results reveal both common and tissue-specific features in hPSCs and descendant hPSC-KCs, and establish an innovative human organoid model for kidney injury and disease. Our findings are broadly relevant for functional studies of human microphysiology, pathophysiology and regenerative medicine.

## Results

### hPSCs form cavitated spheroids in 3D culture

**Figure 1:**
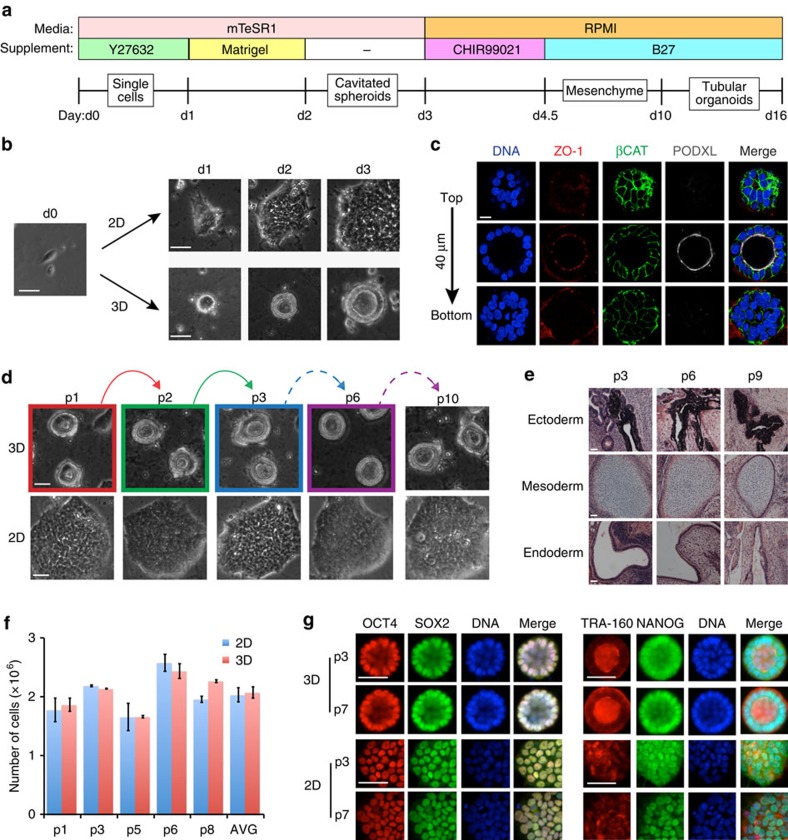
hPSCs form cavitated spheroids in 3D culture. (**a**) Schematic of spheroid-to-organoid culture protocol. (**b**) Phase contrast images of ESCs in sandwich (3D) or monolayer (2D) cultures. Consecutive days are shown, with d0 indicating the time point immediately before sandwiching. (**c**) Confocal optical sections showing PODXL, ZO-1 and βCAT immunofluorescence through a representative spheroid with cavity. Vertical distance from top to bottom row is shown at left. (**d**) Representative brightfield images of hPSCs in 3D cultures that were dissociated (coloured frames) and passaged (matching coloured arrows). Dashed arrows represent serial passages in the 3D condition. Lower row shows cells plated into 2D cultures from dissociated spheroids from each passage. (**e**) Hematoxylin and eosin-stained sections of teratomas generated from hPSC serial 3D passages p3, p6 and p9 showing pigmented epithelium (ectoderm), cartilage (mesoderm) and glandular epithelium (endoderm). (**f**) Cell number (average of duplicate counts for each time point, or AVG of all five time points shown in the last column) in 2D and 3D cultures 72 h after plating. (**g**) Representative immunofluorescence images showing OCT4 and sex-determining region Y box-2 (SOX2) or tumor rejection antigen 1–60 (TRA-1-60) and NANOG localization in p3 and p7 serially sandwiched hPSCs. Scale bars, 100 μm. Error bars, s.e.m.

To evaluate the tissue-specific functions of undifferentiated hPSCs and descendant hPSC-KCs, we developed an adherent, 3D culture system for hPSCs that first produced epiblast spheroids and subsequently kidney tubules (Fig. 1a). Dissociated, undifferentiated hPSCs sandwiched between two layers of dilute Matrigel (0.2 mg ml^−1^) formed compact, ball-like colonies; by 48 h, these formed internal cavities (Fig. 1b). Mature spheroids consisted of a simple columnar epithelium surrounding a hollow lumen (Fig. 1b,c). Spheroid cells exhibited polarized localization of podocalyxin (PODXL) to the luminal surface, zonula occludens 1 (ZO-1) to apical cell–cell junctions, and β-catenin (βCAT) to primarily basolateral membranes (Fig. 1c). Similar apicobasal polarization patterns were observed in monolayer hPSCs (Supplementary Fig. 1a).

We further tested hPSC spheroids for pluripotency and self-renewal, which are the key functional characteristics of undifferentiated hPSCs. In nine serial passages, dissociated cavity-lining spheroid cells generated new cavitated spheroids after sandwiching, or alternatively flat colonies when the final passage was into monolayer conditions (Fig. 1d). Even after extensive serial passages to/from 3D culture, cavity-lining cells implanted into immunodeficient mice efficiently produced teratoma tissues derived from all three embryonic germ layers (Fig. 1e). Two-dimensional (2D) and 3D cultures exhibited similar growth rates, and pluripotency markers continued to be expressed in identical patterns in serially sandwiched cells, including octamer-binding transcription factor 4 (OCT4), sex-determining region Y box-2 (SOX2), NANOG and TRA-1-60 (Fig. 1f,g). 2D colonies and 3D spheroids in pluripotency-sustaining (mTeSR1) media also had nearly identical global gene expression profiles (Supplementary Fig. 1b). Thus, spheroids represented undifferentiated, self-renewing, pluripotent stem cells rather than a differentiated subtype.

During the development of humans and many other mammals, the ICM of the early embryo differentiates into the epiblast, from which all somatic cells are derived. We hypothesized that hPSC spheroids model the epiblast epithelial mass, which forms a columnar epithelium surrounding an early amniotic cavity in human and primate implantation-stage embryos^15^,^16^. Conversely, hPSCs resembling the more primitive ICM were predicted not to cavitate^8^,^12^,^13^. Indeed, ‘naive’ hLR5 iPSCs, which form compact, ICM-like colonies similar to mouse (m)ESCs^8^, did not form lumens in 3D cultures even after 5 days of growth, whereas ‘primed’ hLR5-derived (LD-)iPSCs, which resemble epiblast-stage hPSCs, formed cavities efficiently in sandwich cultures (Supplementary Fig. 2a,b). Both naive and primed hPSCs continued to express nuclear OCT4 and SOX2 in both 2D and 3D cultures (Supplementary Fig. 2c). Similarly, naive mESCs, which resemble the ICM, did not form cavities under a variety of 3D culture conditions, whereas epiblast-stage mEpiSCs formed rosettes surrounding small lumens (Supplementary Fig. 2d–f). These experiments identified the epiblast stage as a critical window for hPSC spheroid lumenogenesis, whereas naive (ICM-stage) hPSCs could not form lumens.

### CHIR99021 differentiates spheroids into tubular organoids

**Figure 2:**
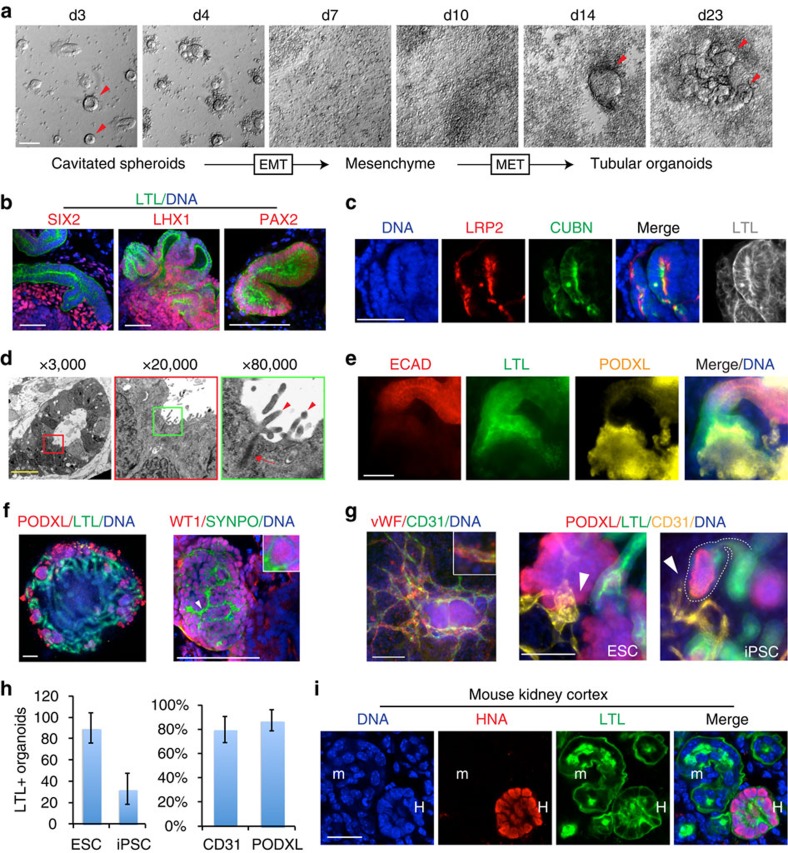
Tubular organoids recapitulate kidney development and architecture. (**a**) Phase contrast images of spheroid differentiation into tubular organoids. Red arrowheads highlight epithelia. (**b**) Confocal optical sections showing LTL with nephron progenitor markers *Sine oculis* homeobox homologue 2 (SIX2), Lin11-Isl1-Mec3 (LIM) homeobox 1 (LHX1), paired box gene 2 (PAX2) and (**c**) proximal tubule markers low density lipoprotein-related protein 2/megalin and cubilin in tubular organoids. (**d**) Electron micrographs of a representative tubule, with progressive magnifications of regions in coloured boxes highlighting apical microvilli (arrowheads) and tight junctions (arrows). (**e**) Wide-field images showing tubule anatomical progression from E-cadherin (ECAD)^+^ to LTL^+^ to PODXL^+^ organoid segments. (**f**) Low-magnification image of organoid with interlacing tubules and peripheral PODXL^+^ aggregates (left) and high-magnification confocal optical section showing co-localization of synaptopodin (SYNPO) and Wilms tumour protein (WT1) in organoid podocyte-like cells (right). (**g**) Wide-field immunofluorescence showing co-localization of CD31 with von Willebrand factor (vWF, left), or with nephron markers in tubular organoids derived from hESCs and iPSCs (right). White arrowheads show interactions between tubular, podocyte-like and endothelial compartments. White dashed outline highlights a representative tubular terminus. Images are representative of one hESC line and three iPSC lines from different patients. (**h**) Number of tubular organoids formed per unit surface area in cultures of hESCs and iPSCs (left) and per cent of these LTL^+^ organoids associated with CD31^+^ and PODXL^+^ cell types within the organoid (right). (**i**) Confocal images of organoid-derived human tubule (H) with LTL reactivity after 3 weeks of growth inside the developing mouse kidney cortex (m). Scale bars, 100 μm. Error bars, s.e.m (*n*≥3 experiments).

To differentiate epiblast spheroids into descendant epithelia, we applied a directed differentiation regimen originally designed for cardiomyocyte generation from 2D cultures, involving the sequential inhibition of glycogen synthase kinase-3β (GSK3β) and wingless-related integration site (WNT) signalling^29^. Remarkably, rather than forming cardiomyocytes, spheroid cells underwent epithelial-to-mesenchymal transition to form a confluent monolayer that by day 10 aggregated into folds and initiated mesenchymal-to-epithelial transition into convoluted, translucent, tubular organoids (Fig. 2a, Supplementary Fig. 3a,b and Supplementary Movie 1). Optimization of this new protocol revealed that spheroid formation, treatment with the GSK3β inhibitor Chiron Technologies 99021 (CHIR99021) and subsequent incubation in B27-supplemented media were sufficient to induce tubular differentiation, whereas insulin and WNT inhibition steps were dispensable (Supplementary Fig. 3c,d; the optimized protocol is shown in Fig. 1a).

We examined these tubular organoids for the expression of kidney markers. *Lotus tetragonolobus* lectin (LTL), a marker of kidney proximal tubules, reacted strongly with tubular structures and appeared enriched in tubular lumens (Fig. 2b,c). By comparison, epiblast spheroids examined side-by-side with tubules had negligible affinity for LTL (Supplementary Fig. 3e). As LTL reacts strongly with kidney tubules and also with certain other epithelia, we performed a more thorough characterization of these organoids with markers of kidney and other organs. Tubules expressed the nephron progenitor/renal vesicle markers Lin11-Isl1-Mec3 (LIM) homeobox 1 (LHX1) and paired box gene 2 (PAX2; Fig. 2b). *Sine oculis* homeobox homologue 2 (SIX2) was expressed in mesenchyme adjacent to the tubules but not in the tubules themselves, consistent with the developmental restriction of this marker to the metanephric mesenchyme (Fig. 2b). The endocytosis receptors low density lipoprotein-related protein 2 (megalin) and cubilin were co-expressed apically and co-localized with LTL in tubule segments (Fig. 2c). Apical microvilli and tight junctions were observed in tubules by electron microscopy (Fig. 2d). We furthermore observed anatomical progression of tubules from segments expressing E-cadherin, a distal tubule marker^30^, to LTL^+^ segments (proximal tubule), to capsule-like structures containing PODXL^+^ podocyte-like cells (Fig. 2e). These PODXL^+^ cells aggregated at the termini of tubules, at the periphery of the organoids (Fig. 2f). They exhibited a spherical and tightly clustered morphology, lacked LTL reactivity, and co-expressed additional markers of kidney podocytes^31^,^32^,^33^, including Wilms tumour protein (WT1) and synaptopodin (Fig. 2f,g). Endothelial cords expressing CD31 and von Willebrand factor also arose within organoids, contacting both tubular and podocyte-like cell populations (Fig. 2g). These results suggested that tubular organoids contained self-organizing hPSC-KC subpopulations capable of nephron-like segmentation and vascular structures.

Using this protocol, H9 hESCs and three different iPSC lines produced organoids incorporating cells and structures with characteristics of proximal tubules, endothelial cells and podocytes, in kidney-like segmental arrangements (Fig. 2g). hESCs showed the highest efficiency of differentiation, yielding ∼90 organoids per 1.9 cm^2^, with LTL^+^ cells representing ∼25% of the total culture (Fig. 2h and Supplementary Fig. 4a). Approximately 80% of LTL^+^ organoids included endothelial (CD31^+^) and podocyte-like (PODXL^+^) cell populations (Fig. 2h). Markers of neuroectoderm (TUJ1) or intestine (CDX2) were absent within organoids; however, separate clusters of TUJ1^+^ cells were observed in the cultures at a ratio of approximately 1:2 to kidney organoids, and projected axon-like processes towards the tubules (Supplementary Fig. 4b–d). RNA and protein analysis during the time course of differentiation revealed sequential induction of markers characteristic of mesendoderm, nephron progenitors and finally proximal tubules and podocyte-like cells (Supplementary Fig. 4e). To assess their potential to engraft *in vivo*, tubular organoid cultures were dissociated and implanted into kidneys of neonatal immunodeficient mice. After 3 weeks, we identified human nuclear antigen-positive (HNA^+^) epithelial structures within the mouse kidney cortex, with LTL intensities comparable to neighbouring mouse tubules (Fig. 2i). We concluded that tubular organoids most likely represented the kidney lineage.

### Organoids recapitulate tissue-specific injury and transport

**Figure 3:**
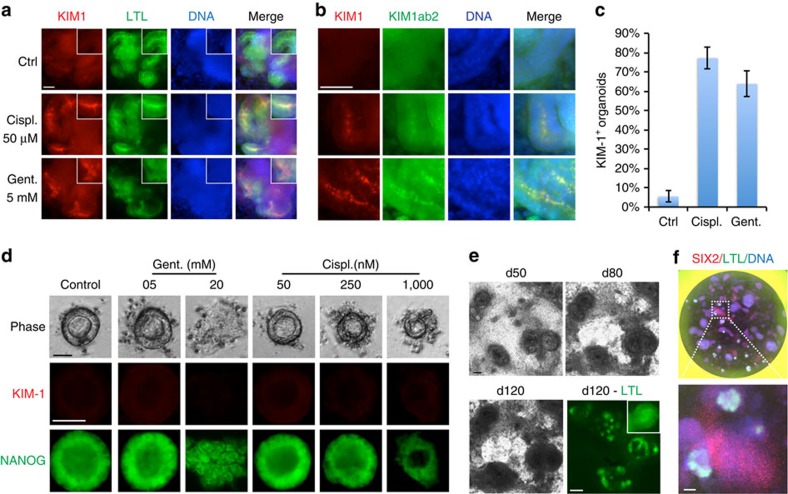
Tubular organoids create a microphysiological model for kidney nephrotoxicity studies. (**a**) Co-localization of KIM-1 antibody AKG7 with LTL or (**b**) with a second KIM-1 antibody (KIM1ab2). (**c**) Quantification of organoids expressing KIM-1 after treatment with 50 μM cisplatin (Cispl.) or 5 mM gentamicin (Gent.), compared with vehicle-treated controls (*n*=3/condition, >50 organoids/experiment). (**d**) Phase contrast of living cells, or KIM-1 and NANOG immunofluorescence, in epiblast spheroids treated with titrations of cisplatin and gentamicin. No KIM-1 expression is observed at any dose including 5 mM gentamicin, a concentration sufficient to induce KIM-1 upregulation in kidney organoids. Cisplatin was toxic to spheroids at concentrations>1 μM. (**e**) Phase contrast time course of tubules in culture. After 120 days of culture (d120), tubules were fixed and stained for LTL. (**f**) Whole well of a 96-well plate showing kidney organoids. Scale bars, 50 μm. Error bars, s.e.m.

Tissue-specific functions or disease phenotypes have not been demonstrated in hPSC-KCs. We therefore investigated the potential of kidney organoids to upregulate a clinical biomarker of proximal tubule injury, kidney injury molecule-1 (KIM-1), also known as T-cell immunoglobulin and mucin domain 1 and hepatitis A virus cellular receptor 1 (refs 34, 35). When treated with the nephrotoxic drugs cis-diamminedichloroplatinum(II) (cisplatin) or gentamicin, KIM-1 immunofluorescence was detected at the luminal surface of tubules in ∼80% of organoids, and was confirmed using two different antibodies (Fig. 3a–c). In contrast, while epiblast spheroids exhibited dose-dependent sensitivity to both cisplatin and gentamicin (disintegration), they did not upregulate KIM-1 after treatment, indicating that this response was specific to kidney organoids (Fig. 3d). Notably, LTL^+^ tubules remained stable in extended cultures for at least 120 days, and cultures could also be miniaturized to a 96-well format (Fig. 3e,f). Kidney organoids might therefore provide a long-term, high-throughput model system to evaluate human nephrotoxicity.

**Figure 4:**
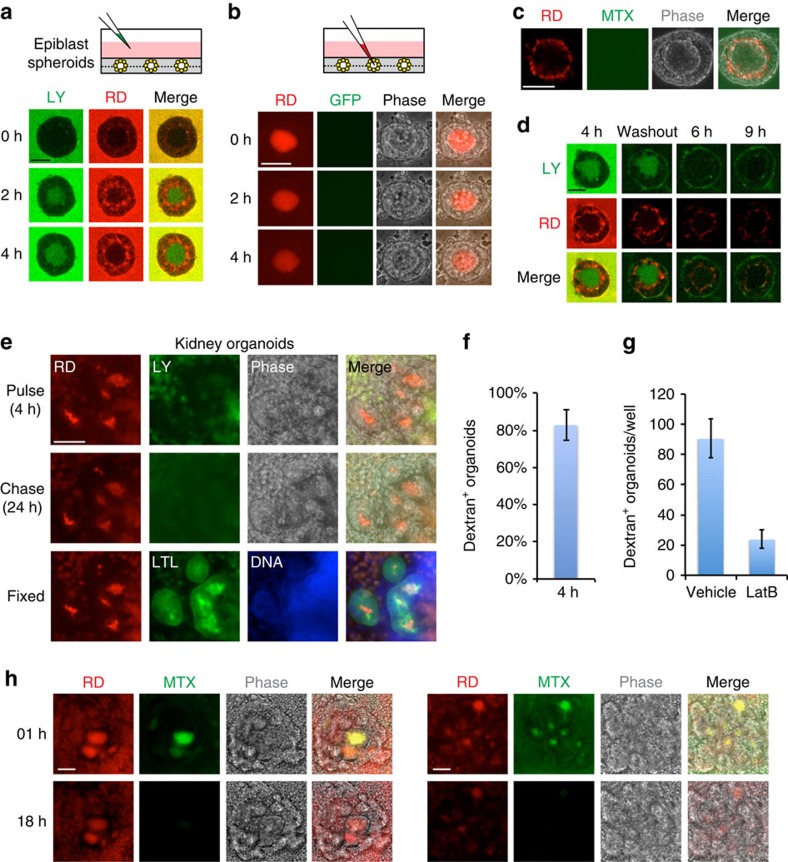
Differential accumulation of fluorescent cargoes in epiblast spheroids versus kidney organoid tubules. (**a**) Experimental schematic and confocal time-lapse images showing hPSC spheroids incubated with lucifer yellow (LY) and rhodamine-conjugated dextran (RD). (**b**) Experimental schematic and wide-field time course of an hPSC spheroid cavity after microinjection with RD. No autofluorescence is detected (green fluorescent protein channel). (**c**) An hPSC spheroid treated with fluorescein methotrexate for 4 h, immediately after washout. (**d**) Time course of an hPSC spheroid incubated with LY or RD molecular dyes for 4 h, after which the media was replaced without dyes (washout). (**e**) Representative time course of a tubular organoid incubated with RD and LY for 4 h (pulse), followed by incubation in fresh media without dyes (chase), fixation and co-localization with LTL. (**f**,**g**) Quantification of tubular organoids that accumulated RD, with or without 2 μM Latrunculin B (*n*=3). (**h**) Fluorescein methotrexate and RD distributions in two representative live organoids 1 h and 18 h after washout. Scale bars, 50 μm (**a**–**d**) or 100 μm (**e**–**h**). Error bars, s.e.m.

To test whether epiblast spheroids and kidney tubules exhibit tissue-specific barrier functions, we developed a real-time assay to visualize molecular diffusion kinetics into and out of lumens, using fluorescent compounds of different sizes. In epiblast spheroids, lucifer yellow (521 Da) added to the culture media for 2–4 h gradually accumulated within cavities, whereas rhodamine-conjugated dextran (RD, 10,000 Da) was excluded from lumens and instead accumulated in apical intercellular regions and formed a bright halo around the lumen (Fig. 4a). Reciprocally, RD microinjected into the cavity remained detectable for hours (Fig. 4b). Another small molecule, fluorescein methotrexate (979 Da), did not accumulate in hPSC lumens (Fig. 4c). When fluorescent compounds were incubated with spheroids for several hours and subsequently washed out, the compounds initially retained their distributions, but faded in intensity over time, indicating that they remained dynamic and were not fixed in location (Fig. 4d). We performed parallel experiments assessing transport of fluorescent macromolecules in differentiated tubular organoids. In contrast to epiblast spheroids, RD localized to tubular lumens in ∼80% of organoids after 2–4 h of incubation, and remained associated during a 24-h washout chase, without corresponding enrichment of lucifer yellow (Fig. 4e,f). Co-incubation with latrunculin B, an inhibitor of actin polymerization and endocytosis, significantly reduced RD accumulation (Fig. 4g). Methotrexate also accumulated in kidney tubules, similar to RD, but appeared more dynamic than RD after washout (Fig. 4h). Tubular organoids thus exhibited transport characteristics typical of proximal tubules^36^,^37^ and distinct from those of epiblast spheroids.

### Podocalyxin promotes epiblast cavitation

**Figure 5:**
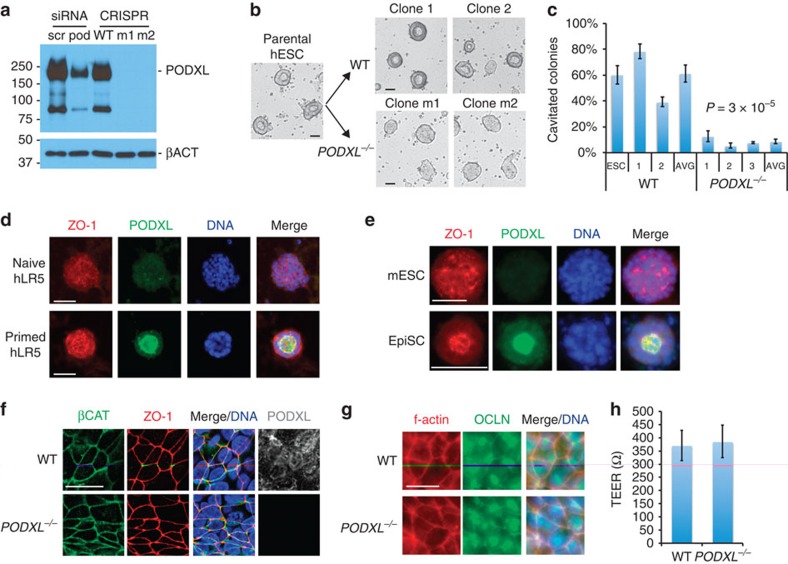
Podocalyxin promotes lumenogenesis in epiblast spheroids. (**a**) Immunoblot for podocalyxin protein in two representative *PODXL*^−/−^ hPSC mutant clones (m1 and m2), compared with CRISPR/Cas9 non-mutant wild-type clones (WT) or cells subjected to scrambled (scr) or podocalyxin (pod) siRNA knockdown. (**b**) Brightfield images of sandwiched parental ESCs were compared with two mutant or two WT CRISPR/Cas9 clones. (**c**) Cavitated spheroids as a percentage of total colonies. Data from pools of WT or mutant cell lines were averaged to determine group means (AVG, *n*≥9) and *P* values. (**d**) Podocalyxin and ZO-1 immunofluorescence in naive and primed hLR5 hPSCs or (**e**) mESCs and EpiSCs. (**f**) Confocal z-sections of undifferentiated hPSCs showing localization of ZO-1 and βCAT in unmodified (WT) or *PODXL*^−/−^ colonies. (**g**) Filamentous actin (f-actin) and occludin (OCLN) immunofluorescence in undifferentiated WT or *PODXL*^−/−^ clones. (**h**) Averaged TEER measurements in WT or *PODXL*^−/−^ monolayers (*n*≥3). Scale bars, 50 μm or (**f**,**g**) 20 μm. Error bars, s.e.m.

Podocalyxin is an apical sialomucin expressed highly in both epiblast and kidney podocytes^1^,^11^,^26^,^27^,^38^,^39^. To investigate the functional role of podocalyxin in these cell types, podocalyxin knockout (*PODXL*^−/−^) hPSCs were generated using the clustered regularly interspaced short palindromic repeats (CRISPR)/Cas9 genome editing system (Supplementary Fig. 5a). Immunoblots for podocalyxin revealed two major bands at ∼220 and 80 kDa that were completely absent in *PODXL*^−/−^ hPSCs (Fig. 5a). TRA-1-60 and TRA-1-81, two pluripotency markers associated with podocalyxin, were ∼40% reduced in knockout cells (Supplementary Fig. 5b). *PODXL*^−/−^ hPSCs exhibited teratoma formation, growth rates and 3D colony size indistinguishable from otherwise isogenic, unmodified hPSCs, indicating that they remained pluripotent and self-renewing (Supplementary Fig. 5c–e). Strikingly, compared with unmodified controls of otherwise identical genetic background, *PODXL*^−/−^ hPSCs in 3D sandwich cultures exhibited a drastic (∼85%) decrease in their ability to produce hollow lumens, appearing instead as solid spheroids by phase contrast microscopy (Fig. 5b,c). Furthermore, podocalyxin expression was much higher in epiblast-stage hPSCs and mEpiSCs, which formed podocalyxin-lined cavities, than in ICM-stage cells, which did not form cavities (Fig. 5d,e). These results revealed that podocalyxin was dispensable for the maintenance of pluripotency but required for epiblast spheroid lumenogenesis.

Podocalyxin is proposed to regulate lumenogenesis through tight junction organization^39^,^40^. In *PODXL*^−/−^ hESCs, however, the junctional components ZO-1, occludin and filamentous actin appeared properly localized, and transepithelial electrical resistance (TEER) was indistinguishable from wild-type controls (Fig. 5f–h). Although lumens were only very rarely observed by phase contrast microscopy in living cells, immunofluorescence in fixed cultures revealed small, compressed lumens in *PODXL*^−/−^ hESC spheroids, lined with apically polarized ZO-1 (Supplementary Fig. 6a). Lucifer yellow added to the media accumulated within these small lumens, while RD was excluded from lumens and accumulated in intercellular foci, similar to wild-type cells (Supplementary Fig. 6b). These experiments demonstrated that podocalyxin is not required to establish polarized, functional tight junctions in hPSCs. To investigate whether podocalyxin might contribute directly to luminal expansion via intermolecular charge repulsion, hPSCs were treated with a low concentration (8 μg ml^−1^) of protamine sulfate, a positively charged polycation that neutralizes the negatively charged, sialylated extracellular domain of podocalyxin^41^. Protamine sulfate strongly inhibited 3D cavity formation, causing mislocalization of podocalyxin to dispersed patches (Supplementary Fig. 6c,d). Collectively, these results revealed that podocalyxin can promote epiblast lumenogenesis independently of its association with tight junctions, likely through a direct, charge-mediated mechanism.

### Podocalyxin regulates junctions in podocyte-like cells

**Figure 6:**
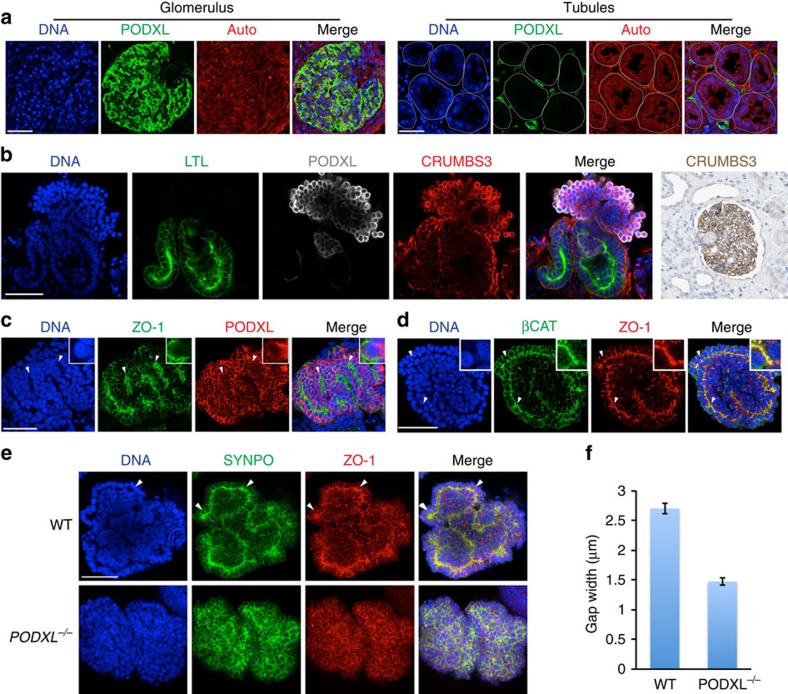
Junctional complexes are disrupted in hPSC-derived *PODXL*^−/−^ podocyte-like cells. (**a**) Confocal optical section of adult human kidney. Podocalyxin is expressed in podocytes and peritubular capillaries, but is absent from tubules (white dotted lines). Auto, autofluorescence. (**b**) Crumbs3 expression in hPSC-derived kidney organoids (confocal red channel) and human kidney tissue (far right panel, immunohistochemistry). (**c**) Confocal optical sections showing distributions of ZO-1 with podocalyxin or (**d**) βCAT in hPSC-derived podocyte-like cell clusters. Arrowheads highlight tracks of junctional complexes between podocyte-like cells. (**e**) Confocal sections of wild-type or *PODXL*^−/−^ podocyte-like cell clusters in tubular organoids. (**f**) Gap widths between adjacent podocyte-like cell nuclei in these cell lines (*n*≥100 gaps pooled from two experiments). Scale bars, 50 μm. Error bars, s.e.m.

We next investigated podocalyxin function in human kidney cell types. In tissue sections from adult human kidneys, podocalyxin was highly expressed in the glomeruli, but was not detected in the tubules (Fig. 6a). Similarly, in hPSC-derived kidney organoids, only podocyte-like cells expressed high levels of podocalyxin (Fig. 6b). We determined the localization pattern of podocalyxin and junctional markers in these cells by confocal microscopy. Both podocalyxin and another podocyte marker, Crumbs3, brightly coated the plasma membrane on the exterior of podocyte-like aggregates, whereas ZO-1, SYNPO and βCAT co-localized in a reciprocal pattern, forming internal zipper-like tracks between adjacent cell layers (Fig. 6c,d). The combined expression of PODXL, Crumbs3, ZO-1, SYNPO, βCAT and WT1 (see Fig. 2f) is not known to occur in any population other than kidney podocytes, nor would such cells be expected to appear alongside LTL^+^ tubular cells in other organs^26^,^27^,^42^,^43^,^44^. These results furthermore reveal that hPSC-KC podocyte-like cells form polarized domains segregating junctional components such as Crumbs3 from ZO-1, consistent with biochemical and microscopic analyses of podocytes *in vivo*^31^,^32^,^33^.

To investigate podocalyxin function in human podocyte-like cells, we produced kidney organoids from *PODXL*^−/−^ hPSCs. In contrast to wild-type organoids, in *PODXL*^−/−^ organoids, the appearance of linear ZO-1^+^SYNPO^+^ tracks was strongly reduced, and junctional markers adopted a more diffuse expression pattern (Fig. 6e). The disappearance of these tracks in *PODXL*^−/−^ organoids correlated with a decrease in gap width between adjacent podocyte-like cells, compared with isogenic controls (Fig. 6f). The absence of podocalyxin did not affect the efficiency of hPSC-KC differentiation into tubules (ZO-1^+^LTL^+^) or podocytes (ZO-1^+^SYNPO^+^; Supplementary Fig. 7a). Tubules, which expressed little to no detectable podocalyxin, exhibited no defects in their morphologies, diameters and LTL or ZO-1 expression patterns in *PODXL*^−/−^ organoids (Supplementary Fig. 7a,c). We concluded that podocalyxin is dispensable for kidney organoid differentiation, but is specifically required in podocyte-like cells for proper junctional organization.

### Genome-modified kidney organoids form PKD-specific cysts

**Figure 7:**
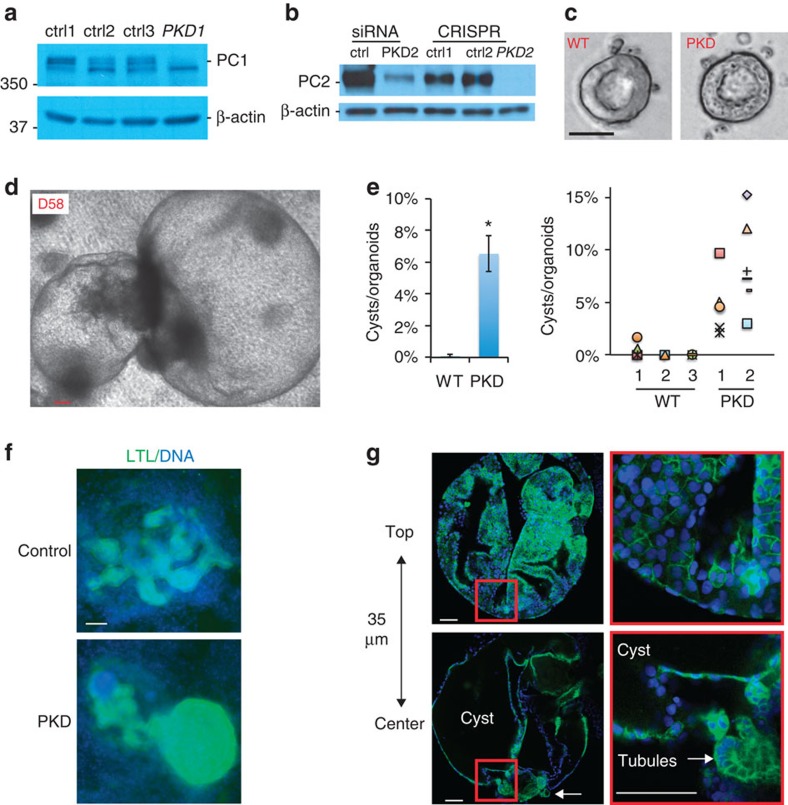
PKD hPSCs model lineage-specific cyst formation. (**a**) Immunoblots showing reduction in full-length polycystin-1 (PC1) in CRISPR-generated *PKD1*^−/−^ hPSCs or (**b**) polycystin-2 (PC2) in *PKD2*^−/−^ hPSCs, compared with isogenic controls. *PKD2* knockdown is shown for comparison. (**c**) Epiblast spheroid morphology in representative PKD knockout hPSCs and controls. (**d**) Representative cyst on day 58 of culture in *PKD2* kidney organoids. (**e**) Quantification of cyst formation rate in PKD knockout organoids and isogenic WT controls as an average of all experiments (*n*>10) or as scatter plots of individual experiments. (**f**) Wide-field epifluorescence images and (**g**) confocal optical sections showing LTL reactivity in cysts. Representative z-sections show hollow center of cyst and associated tubular organoid (arrow). Zoom is shown of red boxed regions. Scale bars, 100 μm. Error bars, s.e.m. *P<0.01.

Lastly, we investigated the potential of kidney organoids to functionally model PKD, which is characterized by the expansion of kidney tubules to form cysts. Biallelic, loss-of-function mutations in *PKD1* or *PKD2* are proposed to contribute strongly to PKD cystogenesis^22^,^23^,^28^. We therefore applied the CRISPR/Cas9 genome editing system to introduce biallelic, truncating mutations in *PKD1* or *PKD2* in hPSCs (PKD hPSCs). Chromatogram analyses and immunoblotting confirmed frame-shift mutations at the target site and demonstrated the absence of the corresponding full-length proteins (Fig. 7a,b and Supplementary Fig. 8a,b). PKD hPSCs differentiated into teratomas similar to isogenic controls, and formed epiblast spheroids with similarly sized lumens (Fig. 7c and Supplementary Fig. 8c,d). These experiments revealed that *PKD1* and *PKD2* were dispensable for the maintenance of pluripotency, and did not detectably affect epiblast spheroid cavitation or morphogenesis.

To test whether these lines might produce phenotypes relevant to PKD in the kidney lineage, we cultured renal organoids derived from PKD hPSCs for several weeks side-by-side with isogenic, unmodified controls. Remarkably, in the PKD hPSC cultures, we observed formation of large, translucent, cyst-like structures alongside tubular organoids (Fig. 7d). These structures remained tethered to the underlying matrix, but moved freely in response to vibration, in contrast to neighbouring tubular organoids that remained fixed in position near the surface of the dish (Supplementary Movie 2). These cysts were detected at a low rate, at ∼6% of that of kidney organoids (Fig. 7e). Importantly, isogenic control hPSCs plated and differentiated side-by-side did not form cysts under these conditions (Fig. 7e). No differences were observed in the overall efficiency of organoid differentiation between PKD hPSCs and controls (Supplementary Fig. 8e). PKD cysts first became noticeable about 35 days after the initial plating and continued to expand for the duration of the culture. Cysts exhibited a strong affinity for LTL comparable to neighbouring tubules (Fig. 7f) and were observed to arise from tubular structures in time-lapse movies (Supplementary Movie 3). Confocal microscopy indicated that cyst-lining epithelia surrounded hollow interior compartments devoid of cells (Fig. 7g). These findings suggested that PKD mutations resulted in aberrant cystogenesis from tubular organoids, in contrast to epiblast spheroids.

## Discussion

The epithelial characteristics of hPSCs and derived kidney cells are poorly understood. Reconstitution of epithelial physiology and morphogenesis in these cell types is important for advancing their potential as human laboratory models and regenerative therapeutics. The described culture system and assays establish a framework for generating and functionally profiling undifferentiated hPSCs and descendant hPSC-KCs in three dimensions. hPSCs are a well-characterized, homogenous and genetically diverse cell type that includes patient-specific, immunocompatible iPSCs^45^,^46^. Well-functioning hPSC-derived epithelia may therefore have applicability for regenerative medicine.

**Figure 8:**
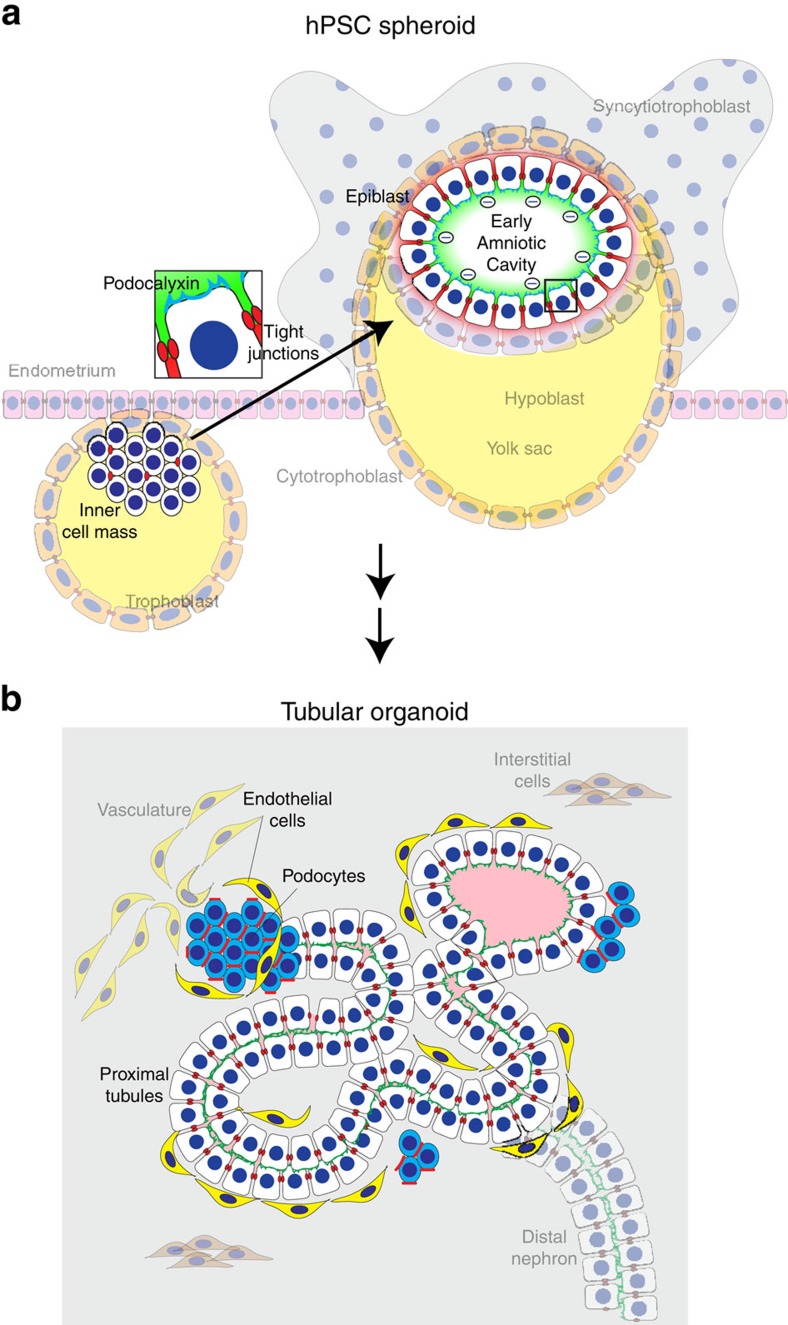
Models of hPSC-derived epithelia. (**a**) Model of hPSC lumenogenesis. ICM-stage hPSCs lack polarized tight junctions, resulting in randomized aggregate formation. Epiblast-stage hPSCs are polarized with continuous tight junctions, and thus organize into a single-cell epithelium. In 3D growth, polarized accumulation of podocalyxin (blue) at the apical membrane results in charge repulsion (negative charges), promoting separation of the cells to form a lumen. Tight junctions (red) permit the entry of small (green) molecules but exclude macromolecules (red), which accumulate in intercellular spaces. Zoom of boxed area is shown, highlighting polarized epithelial cells. (**b**) Architecture of a proximal tubule within a kidney organoid. An elongated proximal tubule forms a simple columnar epithelium which binds LTL (green) on the apical surface. Surrounding podocyte-like cells express high levels of podocalyxin and form a less organized, aggregate structure at tubular termini. ZO-1 (red) is expressed at a sub-apical position, restricted by podocalyxin (blue). Endothelial cells interact closely with both tubular and podocyte-like compartments. Faded background structures place epithelial structures formed *in vitro* into their proposed context *in vivo*.

We demonstrate, for the first time, that undifferentiated, epiblast-stage hPSCs form cavitated spheroids in 3D culture, similar to rosettes recently derived from mESCs, but with expanded lumens^11^. By directly comparing 2D and 3D cultures, our studies reveal that spheroid formation at the epiblast stage can significantly affect subsequent cell fate decisions, producing tubular organoids instead of cardiomyocytes. These organoids recapitulate key characteristics of kidney development and physiology *in vitro*, which have been challenging to model using primary adult or embryonic kidney cells^1^,^47^,^48^,^49^. In contrast to previous protocols for kidney directed differentiation from hPSCs^18^,^19^,^20^,^21^, our simple, two-step procedure of spheroid formation followed by GSK3β inhibition in growth-factor reduced Matrigel does not require exogenous supplementation with fibroblast growth factor 2, activin or bone morphogenetic protein. The tubular structures are surrounded by dilute extracellular matrix in an adherent, microplate format that is experimentally accessible, scalable and potentially high throughput^18^,^19^,^20^,^21^. These structures exhibit a lineage complexity that differs from conventional kidney cell lines and organoids^1^,^47^. All the major components of the developing proximal nephron—tubular cells, endothelial cells, nephron progenitors and podocyte-like cells—are represented within each individual organoid, in kidney-like architectures (Fig. 8b). The proximal tubules transport fluorescent cargoes in a characteristic manner, which is distinct from the pluripotent spheroid epithelia from which they derive^36^,^37^. When injured, tubules express a clinical biomarker, KIM-1, a response that is highly characteristic of the proximal tubule *in vivo* but lost in de-differentiated primary cultures^34^,^35^. This may provide a quantifiable human standard with which to predict proximal tubule nephrotoxicity, a frequent cause of failure in drug development.

As our studies of *PODXL*^−/−^ hPSCs illustrate, this advanced differentiation system can be combined with CRISPR/Cas9 genome editing to determine the function of specific genes in different human cell types, on an isogenic genetic background. Our results suggest a molecular model whereby the combination of apicobasal polarization, tight junction organization and podocalyxin expression distinguishes epiblast-stage hPSCs from ICM-stage progenitors and promotes formation of the early amniotic cavity (Fig. 8a). Although podocalyxin upregulation and tight junction organization occur simultaneously, tight junctions can organize and function independently of podocalyxin in hPSCs. In contrast, in podocyte-like cells, podocalyxin plays a dominant role in the organization of junctions and the spacing of adjacent cells (Fig. 8b), consistent with reports of tight junction phenotypes during rodent glomerulogenesis and in MDCK cells^26^,^27^,^38^,^39^. Thus, although podocalyxin plays an anti-adhesive role in both epiblast and podocyte, its effect on cell polarity and tight junctions is limited to podocytes. Further investigations are required to determine the precise molecular mechanisms underlying these cell type-specific differences, which suggest the presence of co-regulatory factors present in one cell type but not the other. As alterations in podocalyxin expression and the podocyte cytoskeleton are a well-described characteristic of human glomerular disease states^24^,^25^,^26^,^27^, such studies may produce new insights into pathophysiology and treatment.

PKD is among the most common monogenic diseases and of major interest to both clinicians and cell biologists. Existing cellular systems have reported quantitative differences in the formation of simple spheroids or ‘cysts’ attributed to defects in PKD gene expression^50^,^51^,^52^. However, even wild-type cells frequently form cysts in these systems. A reproducible system for PKD-specific cyst formation from tubules is therefore an important goal for the field, particularly in humans where species–specific pathophysiology and therapy is of clinical interest^17^. We find that loss-of-function PKD mutations result in cyst formation from hPSC-derived tubular cells, which is not observed in isogenic controls^17^,^22^,^23^. This finding suggests that PKD-specific cystogenesis from tubules is a cell-intrinsic phenomenon that can be modelled in a minimal system *in vitro*. As cystogenesis was observed for both *PKD1* and *PKD2* mutants, and was specific to the kidney organoids but not epiblast spheroids, the phenotype is both gene specific and lineage specific in this system. Further studies are required to determine the cellular basis of cystogenesis in this system and whether iPSCs from PKD patients, which have heterozygous mutations and variable genetic backgrounds, also produce cysts^17^. As cysts are a relatively rare phenomenon, improvements in iPSC differentiation efficiencies may be required to perform such experiments.

The described hPSC system does have limitations. For instance, we have not yet observed formation of a vascularized glomerulus from hPSC podocyte-like cells and neighbouring endothelia. The tubules also do not contain a full brush border. Although SIX2^+^ mesenchyme was observed adjacent to tubular cells, we did not observe evidence of ureteric bud markers in these tubules. Rather, the tubules have characteristics of proximal tubules derived from the SIX2^+^ mesenchyme, which was induced to differentiate through a non-developmental pathway. Neurons were abundant in these cultures and might possibly represent a source of inductive signals for kidney tubular differentiation in the absence of ureteric bud, similar to embryonic spinal cord^53^. A further limitation of this hPSC-based system is the lack of widely available fluorescent reporter lines with which to perform lineage tracing experiments. One possible solution to this problem would be to adapt this protocol for mouse EpiSCs, which are similar to hPSCs in phenotype. For instance, EpiSCs from the *Six2-eGFPCre* reporter mouse might be used to determine with greater certainty whether all tubular cells in our system derive from the SIX2^+^ mesenchyme, using developing kidneys from this mouse as positive controls^54^. Overall, our findings suggest that while kidney differentiation is indeed occurring from hPSCs, this process *in vitro* does not fully recapitulate developmental kidney nephrogenesis. Dedicated studies involving fluid flow and the tissue microenvironment *in vivo* are required to further develop this system into fully functional nephrons, for more advanced disease modelling and therapeutic application.

In conclusion, we have developed a 3D culture system that reconstitutes functional, structured epithelia modelling the epiblast, kidney tubular cells and podocyte-like cells. These pluripotent and descendant epithelia share certain key structural features, but they can nevertheless recapitulate stage-specific transport characteristics and morphogenesis mechanisms. This provides an accurate and reproducible platform in which to model human microphysiology, injury and disease at distinct developmental stages. Genome-modified tubular organoids functionally recapitulate kidney disease phenotypes, strengthening the identification of these structures as kidney and establishing innovative cellular systems for studying human renal physiology and pathophysiology *in vitro*. The described methodologies are broadly applicable and adaptable to diverse tissues and genetically diverse backgrounds, and can be used immediately to experimentally investigate molecular pathways relevant to human epithelial diseases. In the longer term, this system may provide a useful setting in which to optimize and test the functionality of patient-derived epithelia *in vitro*, before regenerative graft administration.

## Methods

### 3D Culture

Cell lines included H9/WA09 hESCs (WiCell), iPSCs from BJ foreskin fibroblasts (ATCC CRL-2522) and HDFα human dermal fibroblasts (Invitrogen C-013–5C) previously derived in our laboratory^17^, human-LIF-plus-5-reprogramming-factors (hLR5) iPSCs provided by Niels Geijsen (Hubrecht Institute)^8^, human fibroblast hfib2-iPS4 and hfib2–iPS5 iPSCs provided by George Daley (Boston Children’s Hospital), and mESC lines J1 (ATCC SCRC-1010), R1 (ATCC SCRC-1011), and v6.5 (Novus Biologicals NBP1-41162). Cells were maintained feeder-free on 3% Reduced Growth Factor GelTrex (Life Technologies) for at least one passage in the following media: hPSCs in Modified Tenneille’s Special Recipe 1 (mTeSR1, StemCell Technologies); mESCs in N2/B27 medium+2i (0.5X Neurobasal media, 0.5X Dulbecco’s Modified Eagle Medium/Nutrient Mixture F12 (DMEM/F12) media, 0.5X N2 supplement, 0.5X B27 supplement, 0.5X Glutamax, 0.0025% bovine serum albumin and 0.1 mM β-mercaptoethanol (all from Thermo Fisher Scientific), 1,000 U ml^−1^ mouse leukaemia inhibitory factor (LIF, Millipore), 3 μM CHIR99021 (Stemgent), and 100 nM Sigma PD173074)^12^; and hLR5 iPSCs in hESC media (DMEM/F12 with 20% knockout serum replacement, 1X non-essential amino acids, 1X Glutamax, 1X penicillin-streptomycin and 0.1 mM β-mercaptoethanol, all from Thermo Fisher Scientific) pre-incubated/conditioned for 48 h by 4.0 × 10^6^ gamma-irradiated mouse embryonic fibroblasts (GlobalStem) per 50 ml and subsequently supplemented with 10 ng ml^−1^ human LIF (Sigma) and 2 ng ml^−1^ doxycycline (Sigma)^8^. Cells were dissociated with Accutase (StemCell Technologies) or TrypLE (Thermo Fisher Scientific). LD-iPSCs were derived from naive hLR5 iPSCs by withdrawing LIF and doxycycline and substituting with 10 ng ml^−1^ fibroblast growth factor 2 (PeproTech)^8^. For thin gel sandwich colonies, cells were plated at 60,000 (primed) or 30,000 (naive) cells/well of a 24-well plate or 4-well chamber slide pre-coated with GelTrex in media supplemented with 10 μM Rho-kinase inhibitor Y27632 (Stemgent). The following day the media was replaced with 500 μl 1.5% GelTrex in mTeSR1. Media was changed after 24 h. For thick gel cultures, 20,000 (epiblast stage) or 6,000 (naive) cells/well of a 96-well plate were resuspended in 75 μl of either buffered collagen I (containing 10 mM HEPES and 1X DMEM), reduced growth-factor Matrigel (BD Biosciences), or a 1:1 mixture of the two, incubated for 45 min at 37 degrees, and then overlaid with 100 μl of media plus Y27632. For serial passaging in thin gels, colonies with lumens in 3D cultures were dissociated 72 h after plating, replated at a density of 300,000 cells/well within a 6-well plate, and cultured for 72 h in either 2D or 3D conditions before dissociation, cell counting and replating. For suspension, 20,000 dissociated hPSCs were plated in mTeSR1 media in one well of a low-adherence 6-well plate. For all cells, media was changed daily.

### Tubular Organoid Differentiation

In all, 60,000–120,000 H9 hPSCs were plated per 1.9 cm^2^ and sandwiched, sufficient to produce scattered, isolated spheroid colonies. Forty-eight hours after sandwiching, hPSC spheroids were treated with 12 μM CHIR (Stemgent) for 36 h, then changed to RB (Advanced RPMI+1X Glutamax+1X B27 Supplement, all from Thermo Fisher Scientific) and replaced every 3 days thereafter. Alternatively, spheroids were treated with 12 μM CHIR in RB minus insulin (RBNI) for 24 h, RBNI for 48 h, 5 μM inhibitor of WNT production-2 (IWP2, Tocris) for 48 h, RBNI for 48 h and RB every 3 days thereafter, as described for 2D cardiomyocyte differentiation^29^.

### Fluorescence and Electron Microscopy

To fix while preserving 3D architecture, an equal volume of 8% paraformaldehyde was added to the culture media (4% final concentration) for 15 min at room temperature. After fixing, samples were washed in PBS, blocked in 5% donkey serum (Millipore)/0.3% Triton-X-100/PBS, incubated overnight in 3% bovine serum albumin/PBS with primary antibodies, washed, incubated with Alexa-Fluor secondary antibodies (Invitrogen), washed and stained with DAPI or mounted in Vectashield H-1000. Primary antibodies included OCT4 (sc-5279; Santa Cruz; 1:100 dilution), NANOG (RCAB0004PF; Cosmobio; 1:300), brachyury (sc-17745; Santa Cruz; 1:100), TRA-1-60 (MAB4360; Millipore; 1:500), TRA-1-81 (MAB4381; Millipore; 1:500), acetylated α-tubulin (051M4770; Sigma; 1:2,000), ZO-1 (339100; Invitrogen; 1:100), CDX2 (-88; Biogenex; 1:100), AQP1 (AB2219; Millipore; 1:500), WT1 (sc-192; Santa Cruz; 1:100), LHX1 (Developmental Studies Hybridoma Bank; 1:50), mPODXL (AF1556; R&D; 1:500), hPODXL (AF1658; R&D; 1:500), HNA (MAB1281; Millipore; 1:200), SYNPO (sc-21537; Santa Cruz; 1:100), CD31 (555444; BD; 1:500), occludin (71-1500; Fisher; 1:100) and Crumbs 3 (HPA013835; Sigma; 1:200). Stains included fluorescein-labeled LTL (FL-1321; Vector Labs; 1:500) and phalloidin–tetramethylrhodamine B isothiocyanate (P1951; Sigma; 1:1,000) for filamentous actin. Fluorescence images were captured using a Nikon epifluorescence 90-I (upright), Eclipse Ti (inverted), or confocal C1 microscopes. For electron microscopy, structures were scraped from the plate after 5 min of fixation, pelleted at 300*g* for 4 min, and the pellet was gently released by pipetting into 0.15 M sodium cacodylate trihydrate (Sigma) dissolved in water (pH 7.3) containing 4% formaldehyde and 2% glutaraldehyde (Electron Microscopy Sciences), post-fixed with osmium tetroxide solution (Sigma), dehydrated in serial ethanol dilutions (Sigma) and embedded in epoxy resin. Semi-thin sections were cut at 1 mm and stained with toluidine blue (Sigma) to identify tubular structures with apparent lumens by light microscopic examination. Ultrathin sections (75 nm) were cut, mounted on 200 mesh copper grids, counterstained with uranyl acetate and lead citrate stains (Electron Microscopy Sciences), and examined in a JEOL JEM-1010 transmission electron microscope.

### Implantation *in vivo*

Kidney organoid cultures (400,000 cells) were dissociated with accutase, pelleted, resuspended in Advanced RPMI and injected into the kidneys of P0 NOD.CB17-Prkdc^scid^/J mice (Jackson Labs). The mice were sutured and killed 3 weeks later and the kidneys were sectioned. To form teratomas ∼2,000,000 undifferentiated hPSCs were dissociated, resuspended in 50 μl cold Matrigel (BD), and injected dorsally into 8-week-old NOD.CB17-Prkdc^scid^/J mice. Growths were collected 8–10 weeks later. Male and female animals were used. Experiments were performed in compliance with ethical regulations and ARRIVE guidelines and in accordance with protocols approved by the Harvard Medical Area Standing Committee on Animals.

### Permeability Assays

To test permeability, media was supplemented with 20 mM HEPES plus Lucifer Yellow carbohydrazide potassium salt (Invitrogen, 38 μM) and Rhodamine-B isothiocyanate dextran (Sigma, 0.5 μM), and imaged by confocal microscopy. For microinjection, 5 μM rhodamine-conjugated dextran solution in mTeSR1 was diluted 1:1 with Phenol Red Solution (0.5%, Sigma) for visualization. A measure of 2 nl was microinjected via a pulled glass capillary microneedle on a Nanoject-2 micromanipulator, and monitored in real-time by wide-field epifluorescence. To block uptake, Latrunculin B (Sigma) was added simultaneously with RD. To measure TEER, 50,000 hPSCs were plated on 24-well transwell plates (Corning) pre-coated with dilute Matrigel. The media was gently exchanged for 10 days until cells were completely confluent. TEER was measured using an EVOM 2 device (World Precision Instruments).

### KIM-1 Induction

Organoids in identically plated wells of a 24-well plate were treated with increasing concentrations of gentamicin (0.05–15 mM) and cisplatin (0.015–5 mM) for 36–48 h, fixed, and processed for immunofluorescence with KIM-1 antibodies AKG7 (Bonventre laboratory, undiluted hybridoma supernatant) or 1400 (Biogen, 1:100 dilution). Immunofluorescence for KIM-1 was observed at moderate, sub-toxic doses which did not induce gross tubular disintegration.

### RNA Interference

Sixteen hours after plating, hPSCs were transfected with Dharmacon Smartpool siRNAs directed against *PODXL* (Fisher M-010617-01-0005), *OCT4* (M-19591-03-0005), *PKD2* (M-006288-01-0005) or scrambled control (D-001206-13-20) in mTeSR1 without antibiotics. Ten hours later, the media was changed and the cells were either cultured in 2D or sandwiched for 3D culture.

### Cas9/CRISPR Mutagenesis

Constructs encoding green fluorescent protein-tagged Cas9 (Addgene 44719) and a guide RNA (Addgene 64711) targeting the second exon of *PODXL* (5′-GCTACACCTTCACAAGCCCGGGG-3′), the first exon of *PKD2* (5′-GCGTGGAGCCGCGATAACCCCGG-3′), or the thirty-sixth exon of *PKD1* (5′-GTGGGTGCGAGCTTCCCCCCGGG-3′) were transiently transfected into H9 hESCs, and green fluorescent protein-expressing cells were isolated by flow cytometric sorting, clonally expanded and screened for clones with biallelic loss-of-function indels. Approximately 200,000 sorted hESCs were plated per well of a 6-well plate in hESC-conditioned mTeSR1 plus Y27632. The media was replaced the following morning without Y27632 and cells were clonally expanded and the *PODXL* gRNA region was amplified by PCR. Chromatogram sequences were analysed manually and mutations were confirmed by immunoblot and immunofluorescence.

### Transcriptome Profiling

hPSCs plated in 2D or 3D were prepared side-by-side using the RNEasy Mini Kit (Qiagen). Samples were subjected to quality control analysis on an Agilent 2100 Bioanalyzer to check for high integrity samples. Qualifying samples were then prepped using the TruSeq stranded mRNA library kit (Illumina). Sequencing was performed on an Illumina NextSeq500 75 × 75 paired end high output run. Samples were aligned to hg19 reference sequence using the TopHat2 splice junction mapper for RNA-Seq reads (http://ccb.jhu.edu/software/tophat/index.shtml) and differential expression calculated using the Cuffdiff program (http://cole-trapnell-lab.github.io/cufflinks/cuffdiff/).

### RT–PCR

RNA was prepared on days 2, 10, 14 and 21 after plating during the differentiation time course using the RNeasy Mini Kit (Qiagen). RNA from all time points was reverse transcribed side-by-side using the M-MLV Reverse Transcription System (Promega). Quantitative RT–PCR reactions were run in duplicate using cDNA (diluted 1:10), 300 nM primers^16^, and iQ SYBR Green Supermix (Bio-Rad) with the iQ5 Multi-Color Real-Time PCR Detection System (Bio-Rad), using β-actin as the housekeeping gene. Primer sequences are as follows: WT1 5′-GGGTACGAGAGCGATAACCA-3′, 5′-TCTCACCAGTGTGCTTCCTG-3′, SIX2 5′-CTGGAGAGCCACCAGTTCTC-3′, 5′-GCTGCGACTCTTTTCCTTGA-3′, LHX1 5′-ATCCTGGACCGCTTTCTCTT-3′, 5′-GTACCGAAACACCGGAAGAA-3′, OCT4 5′-CAGTGCCCGAAACCCACAC-3′, 5′-GGAGACCCAGCAGCCTCAAA-3′.

### Quantification and Statistical Analysis

Quantification was performed to measure differences or similarities between separate cultures detectable by eye. Sample size was chosen based on the researchers’ qualitative assessment of the reproducibility and variability of each particular experiment (higher variability required greater sample size), and the nature of the data being quantified (whole experiment percentage versus metrics of individual structures). The experiments were not randomized. The investigators were not blinded to the conditions during the experiments or analysis. For all experiments, equal numbers of cells were plated. Experimental and control wells (for treatment), fields and samples (for imaging and quantification), and the order of cell lines in plate wells were chosen at random for each experiment. Processing was performed simultaneously and in parallel for all conditions within each experiment. For fluorescence intensity quantifications, images were taken in a single imaging session and at identical exposures and processed identically. The number of cavitated colonies (ellipsoid with lumen) versus flat colonies (non-ellipsoid or without a lumen) was scored manually in phase contrast images of living cells, in which lumens were discerned more easily than in fixed samples. To quantify intensities, line scans of equal length were drawn through randomly selected structures imaged with identical exposures to obtain raw fluorescence values in NIS Elements software (Nikon). The averaged line scan values were plotted with error bars. For CHIR-induced differentiation, ∼6,000 individual cells were identified in low-magnification immunofluorescence images using Cell Profiler 2.0 and fluorescence intensities were measured automatically. Statistical comparisons used a two-tailed *t*-test for two samples with unequal variance (heteroscedastic). Immunoblots were quantified using the ImageJ Gel Analyzer.

## Additional information

**Accession codes:** The RNA-seq data have been deposited in the NCBI BioSample database under accession codes SAMN04105275, SAMN04105276, SAMN04105277, SAMN04105278, SAMN04105279, SAMN04105280, SAMN04105281, SAMN04105282.

**How to cite this article:** Freedman, B. S. *et al*. Modelling kidney disease with CRISPR-mutant kidney organoids derived from human pluripotent epiblast spheroids. *Nat. Commun.* 6:8715 doi: 10.1038/ncomms9715 (2015).

## Supplementary Material

Supplementary Information Supplementary Figures 1-8

Supplementary Movie 1 Brightfield time lapse showing differentiation of spheroids into tubules. Scale bar, 100 µm.

Supplementary Movie 2 Real-time time-lapse movie showing an epithelial cyst grown from PKD2 knockout hPSCs, subjected to vibration.

Supplementary Movie 3 Time-lapse movie of cystogenesis from tubular cells. Days are labeled (d1 = day 19 of the culture).
