# Supplementary material for: Modelling kidney disease with CRISPR-mutant kidney organoids derived from human pluripotent epiblast spheroids
Source: Nat Commun. 2015 Oct 23;6:8715. doi: 10.1038/ncomms9715 (PMC4620584; doi:10.1038/ncomms9715)
Supplement: Supplementary Information — Supplementary Figures 1-8 [file ncomms9715-s1.pdf]

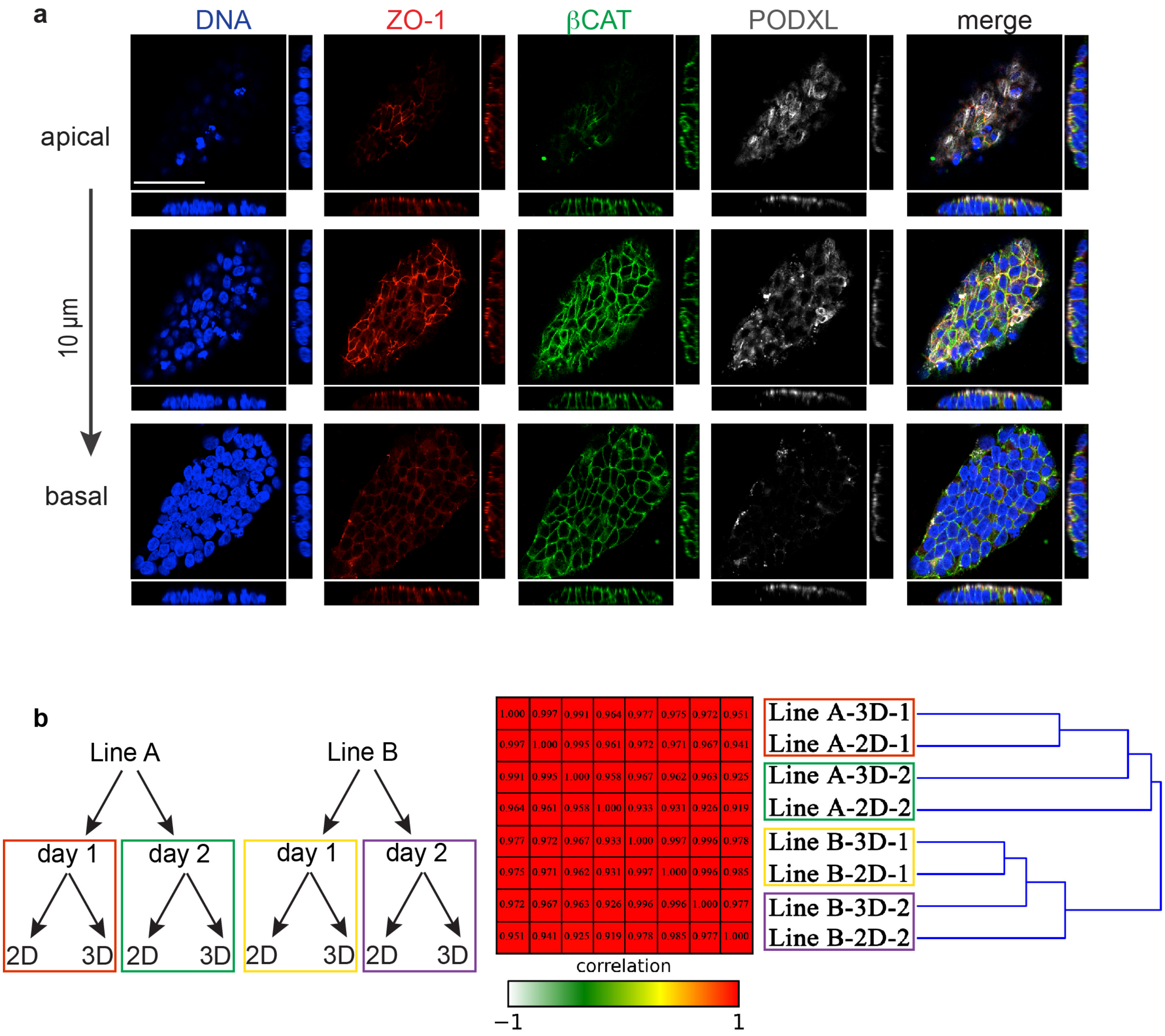

Supplementary Fig. 1. Spheroids resemble monolayer hPSCs. (**a**) Confocal z-stack showing podocalyxin, ZO-1, and β-catenin immunofluorescence through a representative hPSC monolayer colony. Three optical sections through a single colony are shown, from the apical side to the basal side. Podocalyxin is the most apical marker, as shown in the most apical optical section, where it can be seen even in areas of the colony where ZO-1 is not evident. In the middle optical section, ZO-1 appears in a cobblestone pattern at cell-cell contact points. In the most basolateral section, only β-catenin is visible. The sections are accompanied by orthogonal 3D reconstructions of the entire z-stack, which show that ZO-1 is strongly concentrated at areas of cell-cell contact. Vertical distance from top to bottom row is shown at left. (**b**) Schematic of RNA-Seq experiment and resultant hierarchicial clustering of hPSCs in 2D monolayers and 3D spheroids. Two lines are shown (A and B) on two different dates (-1 and -2). Samples in the plot are arranged according to highest correlation as further demonstrated by the dendrogram on the right side. The heatmap grid demonstrates the extent of correlation and is composed of the corresponding R^2 values. Colored boxes in dendogram match groupings in schematic. Scale bars, 50 µm.

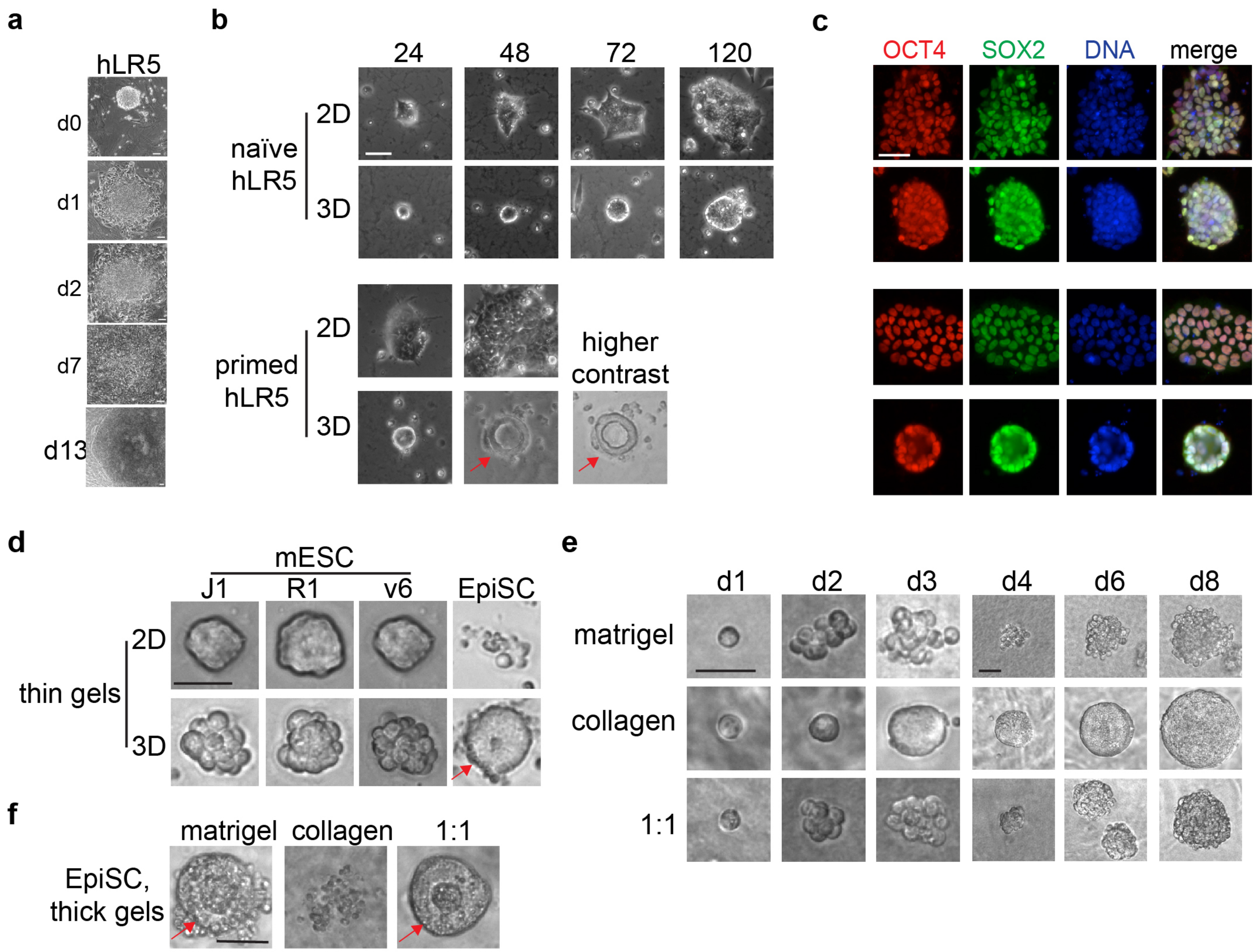

Supplementary Fig. 2. Cavity formation is restricted to the epiblast stage. (**a**) Time course showing transition of naïve hLR5 iPSCs to primed LD-hLR5. (**b**) Naïve and primed hLR5 morphologies in 2D and 3D cultures. (**c**) OCT4 and SOX2 immunofluorescence in naïve and primed hLR5 iPSCs. (**d**) Brightfield morphology of mESCs (lines J1, R1, and v6) or EpiSCs in thin gels 48 hours after sandwiching. Red arrows indicate structures initiating lumenogenesis. Time points (24, 48, etc) represent hours after sandwiching and show different colonies. (**e**) mESC colonies grown from single cells in thick gels of either 100 % Matrigel, 100 % buffered collagen, or a 1:1 mixture of the two (1:1). Similar results were obtained from three separate mESC lines. (**f**) Mouse EpiSCs in thick gels 120 hours after plating. Scale bars, 50 μm.

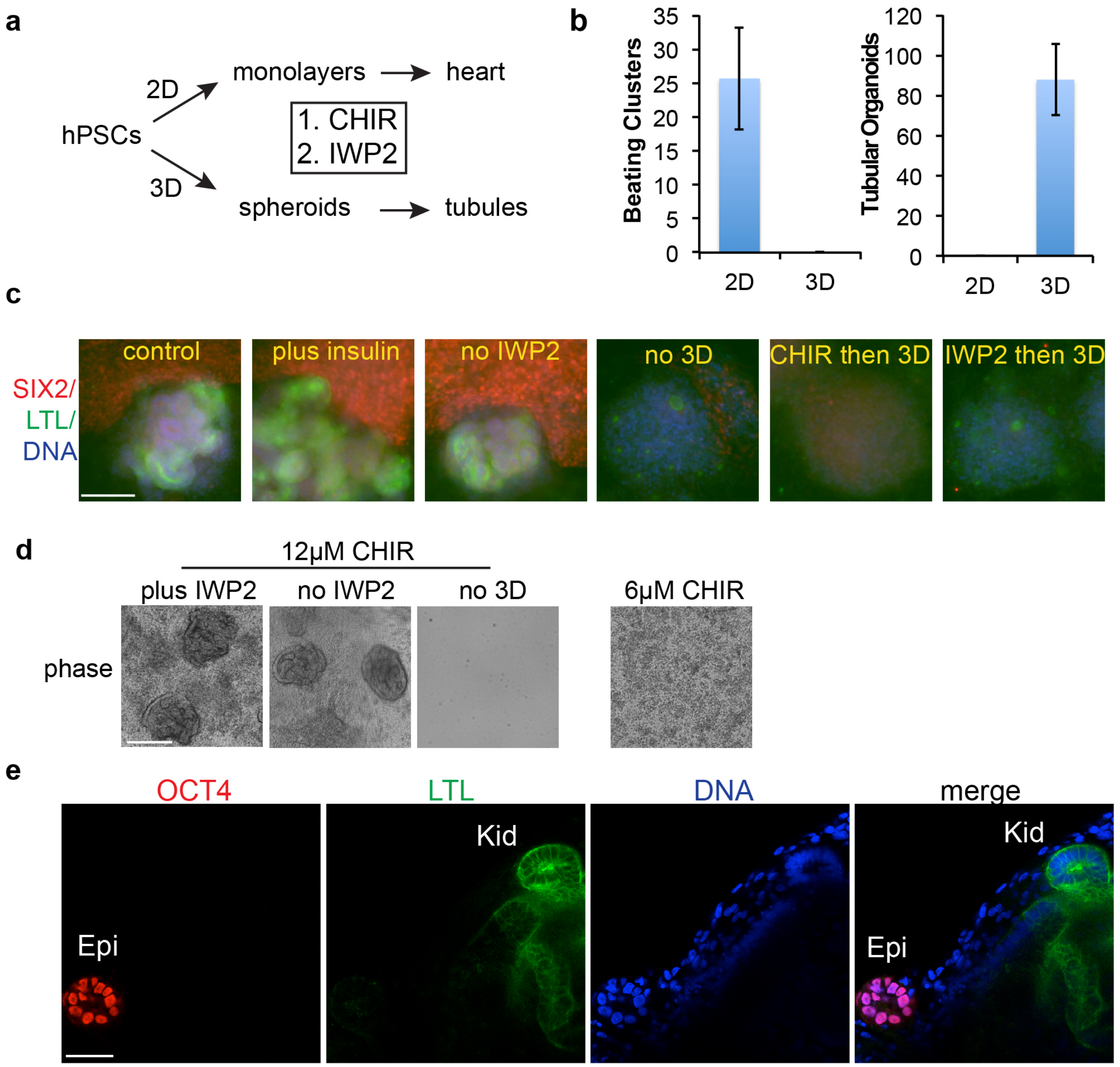

Supplementary Fig. 3. Generation of kidney-like tubular organoids in 3D cultures. (**a**) Schematic of original 2D cardiomyocyte differentiation protocol and application to 3D spheroids. (**b**) Quantification of beating cardiomyocytes and tubular organoids produced in 2D and 3D cultures treated identically with this protocol. (**c**) Day 22 structures representing identically-plated hPSC spheroids treated with the described cardiomyocyte differentiation protocol with the following variations (from left): original protocol (control), use of B27 supplement including insulin, no IWP2 treatment, no sandwiching, sandwiching after CHIR treatment, sandwiching after IWP2 treatment. (**d**) Day 22 structures representing identically-plated cultures treated with CHIR in RPMI with the labeled variations. (**e**) Confocal optical section showing co-localization of OCT4 and LTL in epiblast spheroid (Epi) and kidney organoid (Kid) co-culture. Kidney organoids were picked, transferred to cultures of spheroids from the same iPSC line, and allowed to adhere overnight before fixation and processing for immunofluorescence. Error bars, s.e.m. (n ≥ 3 separate experiments). Scale bars, 100 μm.

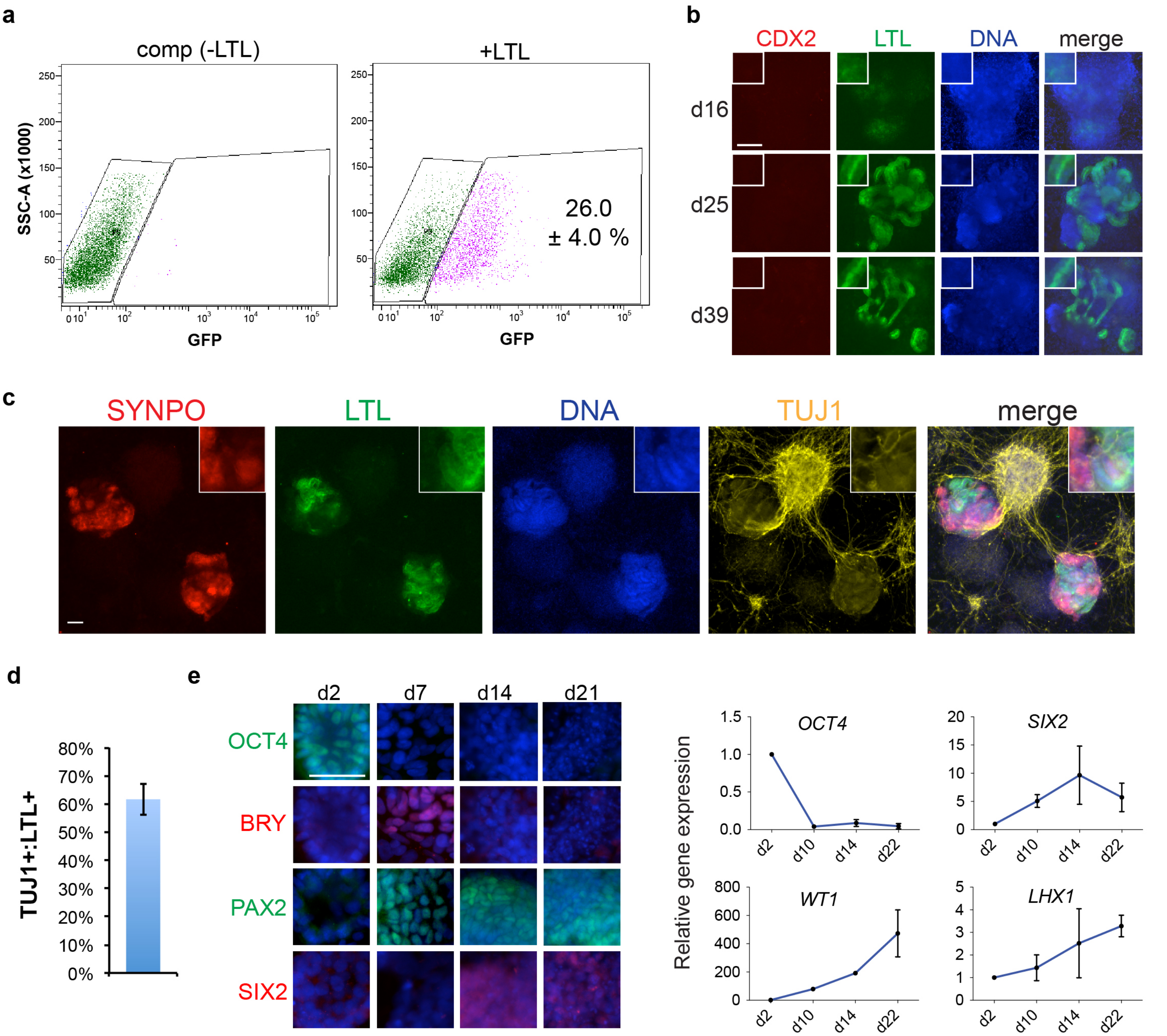

Supplementary Fig. 4. Kidney organoid differentiation. (**a**) Flow cytometry analysis of LTL binding in kidney organoid cells as a percentage of the population. Identically-plated wells of kidney organoids were incubated with LTL or without LTL as a compensation control (comp). Cells were then dissociated and subjected to flow cytometry. The percentage of all cells that were LTL^{+} by FACS is shown (averaged from 3 separate experiments). (**b**)Time course in organoid cultures (rows indicate days of culture) showing absence of LTL co-staining with the intestinal marker CDX2. (**c**) Wide-field low-resolution images showing neural (TUJ1^{+}) clusters and kidney (LTL^{+}) organoids in these cultures. (**d**) Ratio of neural clusters to kidney organoids. (**e**) Immunofluorescence and quantitative RT-PCR time courses sequential expression of stage-specific developmental markers for pluripotency (OCT4), primitive streak (BRY), intermediate mesoderm (PAX2), nephron progenitor (PAX2, SIX2, LHX1, WT1), and podocytes (WT1). d2 represents epiblast spheroids on day 2 after sandwiching, just before CHIR treatment. Error bars, s.e.m. (n ≥ 3 separate experiments and ~30 organoids examined per experiment).

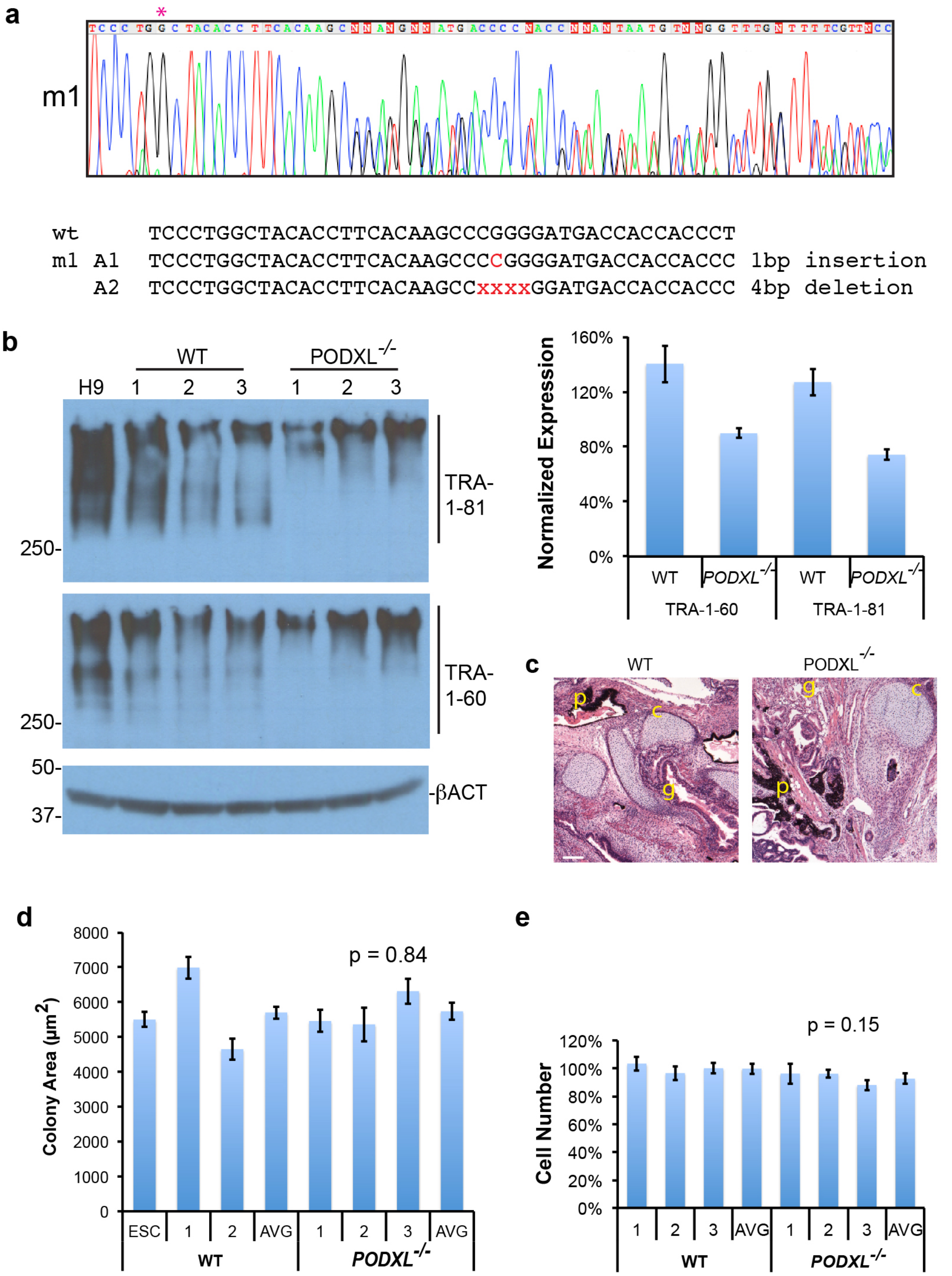

Supplementary Fig. 5. Generation and characterization of *PODXL*$^{-/-}$ hPSCs. (**a**) Chromatogram of a representative CRISPR/Cas9 podocalyxin mutant clone. Pink asterisk marks the beginning of the gRNA sequence. Interpretation shows separated alleles (A1 and A2) aligned against the wild-type sequence. (**b**) Immunoblots showing TRA-1-60 and TRA-1-81 (multiple bands > 250 kDa), with β-actin loading control. Right, quantification of band intensities, normalized to β-actin, averaged from 3-4 clones of wild-type or *PODXL*$^{-/-}$ hPSCs. (**c**) Teratomas from wild-type or *PODXL*$^{-/-}$ hPSCs showing ectodermal pigmented epithelium (p), mesodermal cartilage (c), and endodermal gut-like epithelium (g)**.** (**d**) Total area per epiblast spheroid colony (AVG pooled from ≥ 7 experiments/80 colonies). (**e**) Dissociated cell number after ~7 days growth in escapee (WT) and PODXL-/- hPSCs in 2D culture (AVG pooled from ≥ 14 experiments). Each experiment was normalized to the wild-type. Scale bar, 50 µm. Error bars, s.e.m.

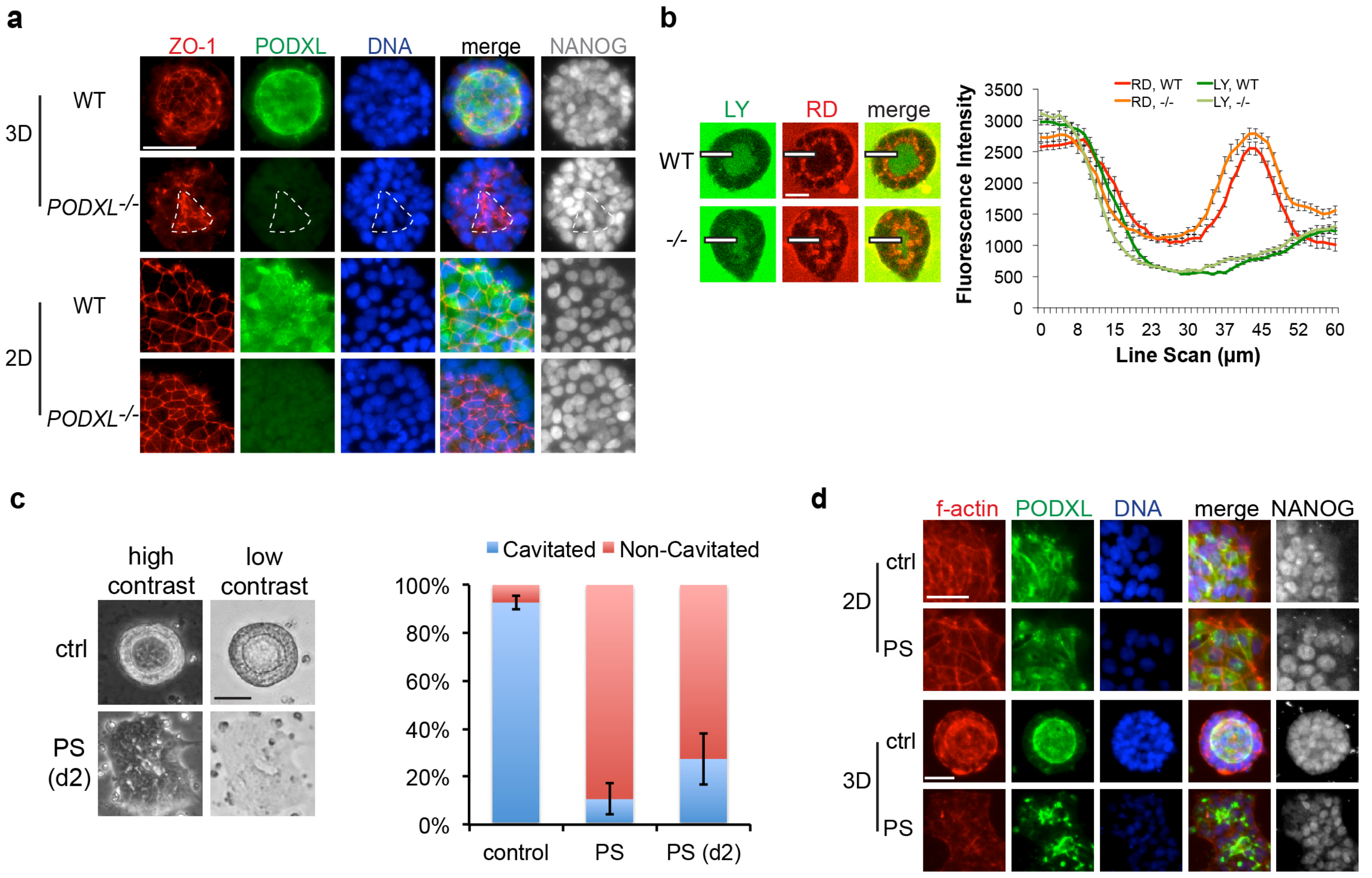

Supplementary Fig. 6. Podocalyxin is dispensable for hPSC tight junction organization. (**a**) ZO-1, podocalyxin, and NANOG immunofluorescence images of CRISPR/Cas9 clones in 2D and 3D culture. A small lumen-like structure is observed in the *PODXL*$^{-/-}$ hPSC 3D aggregate (white dashed lines). (**b**) Representative images (left) and corresponding quantification (right) of wild-type or *PODXL*$^{-/-}$ spheroids after incubation with RD and LY for four hours. Line scans (bordered white rectangles in images) from outside to inside representing 25 wild-type or *PODXL*$^{-/-}$ spheroids pooled from ≥ 2 hESC lines were averaged and plotted. (**c**) Brightfield images (left) and quantification (right) of hPSCs treated with 8 μg/ml protamine sulfate from the time of sandwiching (PS), the day after sandwiching (PS d2), or untreated controls. (**d**) PODXL localization in hPSCs treated with PS. Scale bars, 50 μm.

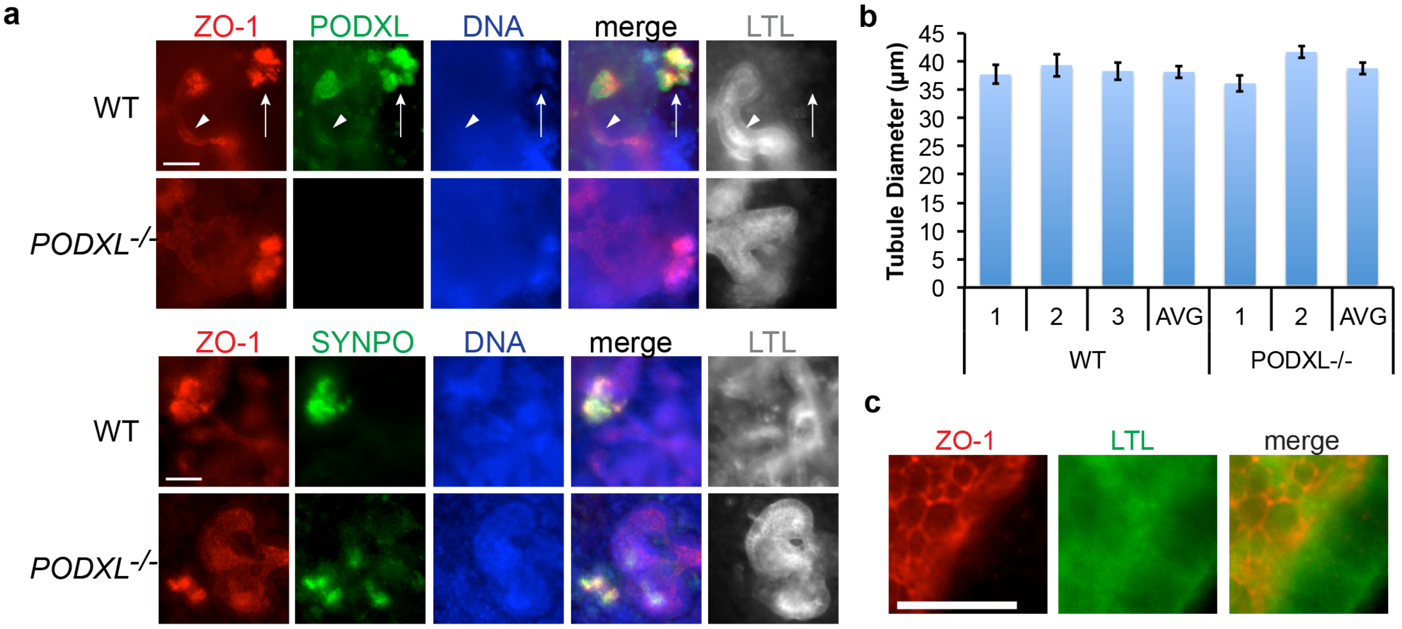

Supplementary Fig. 7. Differentiation of *PODXL*$^{-/-}$ hPSCs into kidney organoids. (**a**) Immunofluorescence for tight junctions (ZO-1), proximal tubules (LTL), and podocytes (PODXL, SYNPO) in tubular organoids derived from *PODXL*$^{-/-}$ hPSCs or isogenic unmodified controls. In controls, podocalyxin is expressed strongly in podocytes (arrows) and faintly in tubules (arrowhead). (**b**) Tubule diameters in wild-type and *PODXL*$^{-/-}$ hPSCs (AVG pooled from ≥ 4 experiments/25 tubules). (**c**) Normal (cobblestone) ZO-1 immunofluorescence in a representative *PODXL*$^{-/-}$ kidney tubule. Scale bars, 50 µm or (c) 20 µm. Error bars, s.e.m.

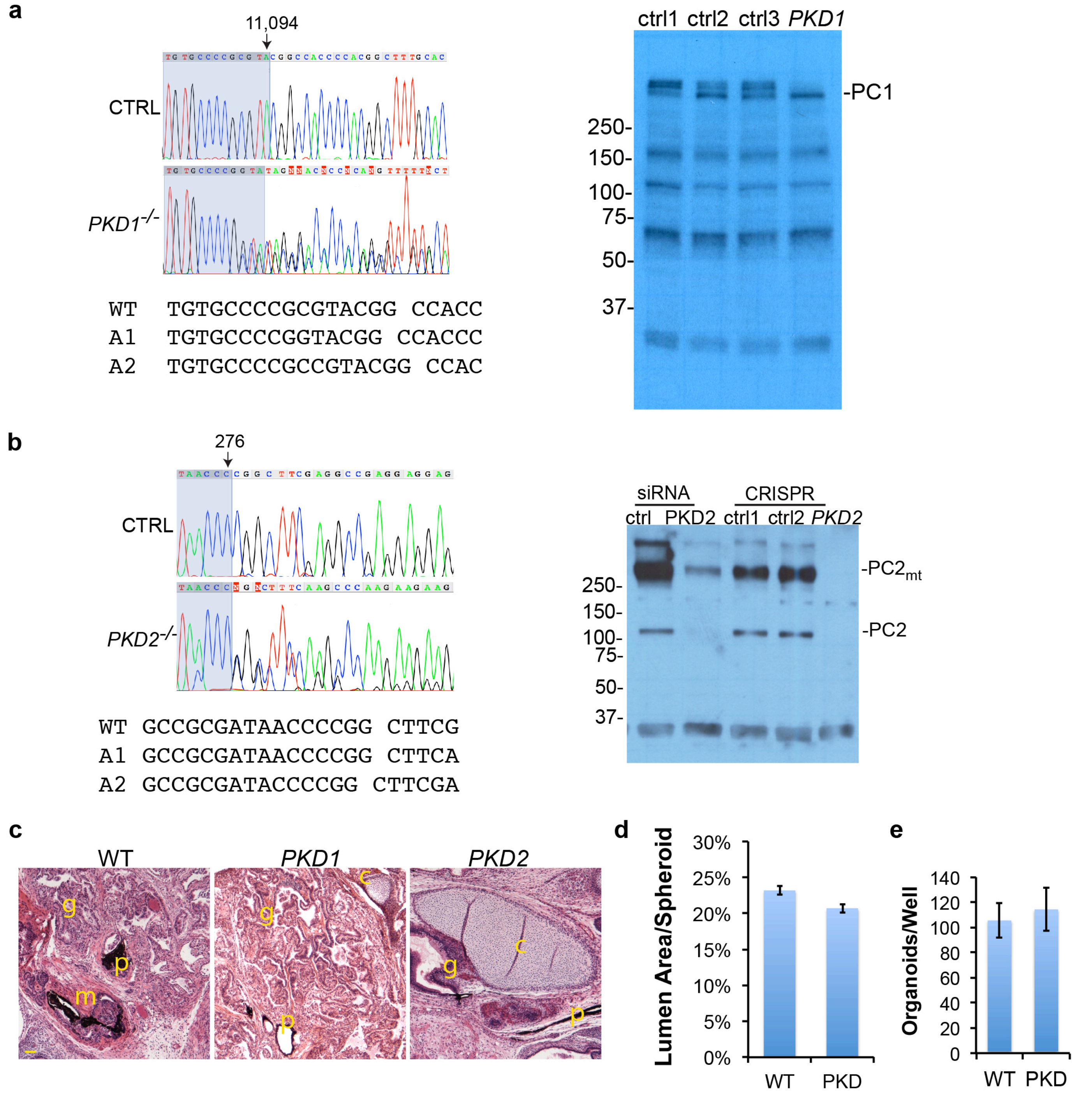

Supplementary Fig. 8. Generation and characterization of *PKD1*$^{-/-}$ and *PKD2*$^{-/-}$ hPSCs. (**a**) Chromatograms (left) and full-length immunoblots (right) of control (CTRL) and *PKD1*$^{-/-}$ hPSCs or (**b**) *PKD2*$^{-/-}$ hPSCs. Shading marks 3' end of gRNA. Arrow indicates base pair number in the coding sequence. Interpretations of allele 1 (A1) and allele 2 (A2) are compared to wild-type consensus sequence (WT). Multimeric PC2 is abbreviated as $PC2_{mt}$. (**c**) Teratomas showing ectodermal pigmented epithelium (p), mesodermal cartilage (c), and endodermal gut-like epithelium (g). Scale bar, 100 µm. (**d**) Quantification of proportional lumen area relative to whole spheroid and (**e**) organoid differentiation efficiency in WT and PKD hPSCs (n ≥ 5 experiments). $p < 0.01$.
